# Pixelated spatial gene expression analysis from tissue

**DOI:** 10.1038/s41467-017-02623-9

**Published:** 2018-01-15

**Authors:** A. Ganguli, A. Ornob, N. Spegazzini, Y. Liu, G. Damhorst, T. Ghonge, B. Thornton, C. J. Konopka, W. Dobrucki, S. E. Clare, R. Bhargava, A. M. Smith, F. Kosari, R. Bashir

**Affiliations:** 1 Department of Bioengineering, University of Illinois at Urbana-Champaign, Champaign, IL 61801 USA; 2 Micro and Nanotechnology Laboratory, University of Illinois at Urbana-Champaign, Champaign, IL 61801 USA; 3 Beckman Institute, University of Illinois at Urbana-Champaign, Champaign, IL 61801 USA; 4 College of Medicine, University of Illinois, Chicago, 60612 USA; 5 Chemical Engineering Department, Tuskegee University, Tuskegee, AL 36088 USA; 6 Feinberg School of Medicine, Northwestern University, Chicago, IL 60611 USA; 7 Mayo Clinic Cancer Center Research, Rochester, MN 55902 USA; 8 Mayo-Illinois Alliance for Technology Based Healthcare, Urbana, IL 61801 USA; 9 Carle Illinois College of Medicine, Urbana, IL 61801 USA

## Abstract

Here, we present a technique that performs on-chip picoliter real-time reverse transcriptase loop mediated isothermal amplification (RT-LAMP) reactions on a histological tissue section without any analyte purification while preserving the native spatial location of the nucleic acid molecules. We demonstrate this method by amplifying TOP2A messenger RNA (mRNA) in a prostate cancer xenograft with 100 µm spatial resolution and by visualizing the variation in threshold time of amplification across the tissue. The on-chip reaction was validated by mRNA fluorescence in situ hybridization (mFISH) from cells in the tissue section. The entire process, from tissue loading on microchip to results from RT-LAMP can be carried out in less than 2 h. We anticipate that this technique, with its ease of use, fast turnaround, and quantitative molecular outputs, would become an invaluable tissue analysis tool for researchers and clinicians in the biomedical arena.

## Abstract (web summary)

Spatial localization of genetic information is important for tissue heterogeneity but difficult to capture with current analytical techniques. Here the authors present “Pixelated RT-LAMP”, an approach that uses parallel on-chip reactions to provide the distribution of target sequences directly from tissue.

## Introduction

The spatial localization of gene expression can unravel important insights into tissue heterogeneity, functionality, and pathological transformations, but the ability to maintain this spatial information remains an enduring challenge in tissue sections routinely used for pathology. Amplification-based spatial gene expression analysis methods provide good sensitivity and specificity but decouple the analyte isolation and biochemical detection steps, making them low throughput and laborious^1–3^. Direct probe-based hybridization techniques such as single-molecule fluorescence in situ hybridization (FISH) allow direct visualization of single RNA molecules in their native cellular context but are not amenable to performing this on tissue section in a high throughput manner. In addition, off-target binding of FISH probes and cellular auto-fluorescence can also become a limiting factor in imaging tissue samples^4–7^. Methods to perform spatially mapped transcriptome analysis on a tissue section can identify multiple targets simultaneously but they must trade-off between the histologic reference and the quality of recovered biomaterials, as staining and manual identification are often needed^8–11^. These constraints limit the translation of the above methods into routine research and clinical practice.

**Fig. 1:**
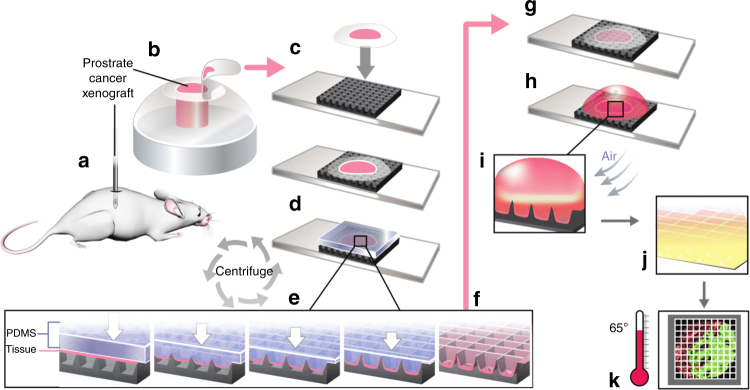
Overall process flow schematic. **a** LNCaP cells are injected into a mouse and prostate cancer xenograft obtained. **b** Xenograft is resected and immediately frozen and embedded in optimal cutting temperature compound (OCT). **c** A 7-µm tissue cryosection is loaded onto our microchip. **d** A cured PDMS block is loaded on top of tissue-chip assembly. **e**, **f** The PDMS shears and partitions the tissue into small pixels at sharp well edges and pushes them into wells under centripetal force in a standard centrifuge. The pixelated tissue adheres to the silanized (APTES) well surfaces and the PDMS is removed. We call this process “tissue pixelation” (time = 2 min). **g** Post pixelation, the tissue is fixed with acetone (time = 10 min). A proteinase K digestion is performed after this to create a pathway for amplification enzymes to reach the target nucleic acids inside the cells (time = 30 min). **h** RT-LAMP reagents are pipetted on chip in bulk (5 µL). **i** Compressed air is blown on it at an angle inside the mineral oil. **j** Excess reagents are sheared away and only fluid inside the wells is retained due to capillary forces. In the above steps, picoliter volume RT-LAMP reagents (~175 pL/well) are loaded onto the chip through a rapid instrument-free technique we call “bulk picoliter reagent loading” (time = 2 min). **k** Quantitative gene expression is visualized through real-time imaging of the amplification reaction in each well performed using only a hot plate at 65 °C and a fluorescence microscope (time = 45 min). Images created by Janet Sinn-Hanlon, The DesignGroup@VetMed, University of Illinois at Urbana Champaign

Here, we introduce a technique which improves upon these drawbacks by analyzing a starting sample of tissue cryosection and performing parallel picoliter reverse transcriptase loop mediated isothermal amplification (RT-LAMP) reactions with minimal sample processing. LAMP is an isothermal reaction which has been shown to be robust against factors in tissue that inhibit a polymerase chain reaction (PCR)^12^. LAMP uses 4–6 primers which identify 6–8 regions on the template for amplification which makes it more specific than PCR^13^. For our work, we designed fingernail-sized silicon oxide-on-silicon chips with an array of 5625 inverted pyramidal wells with ~175 pL volume each and having knife-like sharp distinct edges. As a tissue cryosection is loaded onto our chip, it is partitioned and transferred inside the wells in a process termed “tissue pixelation”. This process divides the solid tissue section into small tissue pixels and takes less than 2 min. This is followed by tissue fixation (10 min), permeabilization (30 min), loading of wells with amplification reagents (2 min), and finally on-chip RT-LAMP reaction on a hot plate (45 min) (Fig. 1). The native spatial distribution of nucleic acid in tissue is preserved throughout the process.

We demonstrate our technique with frozen sections of human prostate tissue xenografts grown in mice. Prostate cancer is the most commonly diagnosed cancer in men and is the second leading cause of cancer death in men in the United States, accounting for more than 25,000 deaths in 2015^14^. The molecular mechanisms fueling prostate cancer pathogenesis remain relatively unknown^15–19^; however, topoisomerase II alpha (TOP2A), a nuclear enzyme involved in chromosome condensation and chromatid separation, has been shown to be upregulated with increasing Gleason score and with hormone insensitivity in prostate carcinoma^20^. Tissue sections from LNCaP prostate cancer cell xenografts in mice were chosen to visualize the spatial variation of TOP2A mRNA using our technique. With a rapid turn-around time of less than 2 h, starting from sample acquisition to RT-LAMP reaction, our technique can perform spatially mapped nucleic acid amplification testing in a typical analytical laboratory.

## Results

### TOP2A mRNA RT-LAMP in a thermocycler

The first step towards our goal was to develop and characterize a sensitive and specific RT-LAMP reaction for TOP2A mRNA. We designed a new RT-LAMP reaction for amplifying TOP2A mRNA using six sequence-specific primers (primer sequences provided in Supplementary Table 1).

**Fig. 2:**
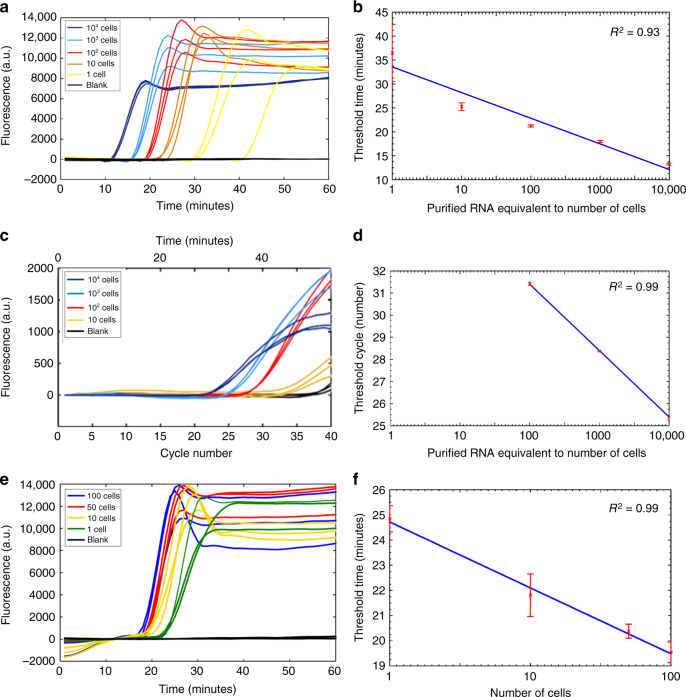
Off-chip RT-LAMP assay characterization. **a**, **b** Amplification curves and standard curve of the TOP2A mRNA RT-LAMP with purified total RNA extracted from LNCaP cells. 10^4^ cells had 940 ng of purified total RNA per reaction as measured with nanodrop spectrophotometer. **c**, **d** Amplification curves and standard curve of the RT-PCR assay for TOP2A mRNA performed using previously published primers^20^. Our RT-LAMP assay can detect TOP2A mRNA from a single cell in a reaction tube, whereas the RT-PCR assay can detect mRNA from only up to 100 cells (~9.4 ng total RNA) in a reaction tube (25 µL per reaction). The amounts of RNA per reaction for each dilution was the same as in RT-LAMP (**a**) to allow direct comparison. **e**, **f** Amplification curves and standard curve of the TOP2A mRNA RT-LAMP assay with whole cells spiked directly into the reaction tubes. TOP2A mRNA down to a single cell could be reliably amplified. All the reactions had three replicates (*n* = 3) and the error bars show the standard deviation (s.d.)

To characterize our assay, RT-LAMP experiments using purified total RNA from xenograft tissue sections and cultured LNCaP cells were performed in a commercial thermocycler and compared with RT-PCR reactions performed using previously published primers with same RNA concentrations^20^. Figure 2a, b and Supplementary Fig. 2 show the amplification curves and the standard curve for RT-LAMP reactions from purified RNA from LNCaP cells and tissue xenograft, respectively. A good linear fit for the standard curves was obtained for both reactions (*R*^2^ = 0.93 and 0.98 for total RNA from cells and tissue, respectively). Figure 2c, d shows the amplification and standard curves for TOP2A qRT-PCR reaction with the same starting RNA as in RT-LAMP. TOP2A RT-LAMP was found to be at least one order of magnitude more sensitive than the corresponding RT-PCR reaction with detection down to a quantity of total RNA equivalent to that of a single cell (in 25 µL tube-based reactions).

Next, to demonstrate the robustness of our RT-LAMP reaction, we spiked 1–100 LNCaP cells directly in a 25 μL reaction tube using hemocytometer counting, and performed RT-LAMP. Figure 2e, f show the amplification fluorescence curves and standard curve for this experiment. Reaction with a single cell could be reliably amplified even in the presence of cell lysate. As seen from Fig. 2a, e, the amplification reaction works better for 100 cells spiked directly into the reaction as compared to purified RNA from 100 cells. We believe that this is due to the inefficiency of the RNA purification process. As will be shown in later sections, in our on-chip experiments on prostate cancer xenograft tissue, we find that the tissue debris remains attached to the bottom of the wells and hence does not interfere with the amplification reaction in the solution. This may have contributed to minimizing the effect of tissue contaminants in the assay.

### Tissue pixelation and bulk picoliter reagent loading

**Fig. 3:**
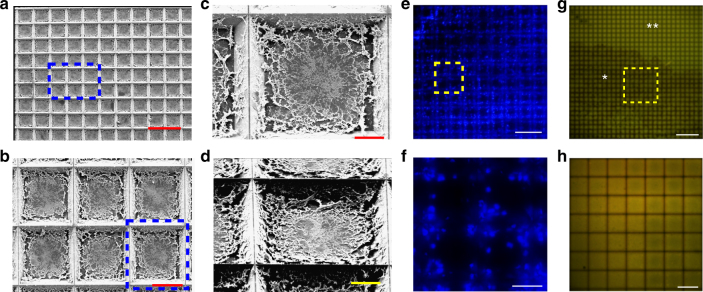
Tissue pixelation and Bulk picoliter reagent loading characterization. **a**–**d** SEM characterization after tissue pixelation. Tissue partitioning and division into small pixels can be clearly visualized as tissue is seen inside the wells. The blue box in **a** is shown in **b** and the blue box in **b** is shown in **c**, **d** (scale bar: **a** 200 µm; **b** 50 µm; **c**, **d** 20 µm). **e**, **f** DAPI-fluorescence imaging of the same pixelated tissue showing nuclei inside the well boundaries. **f** shows the region in yellow box in **e** (scale bar: **e** 200 µm; **f** 50 µm). **g**, **h** Characterization after bulk picoliter reagent loading in tissue-loaded wells. Fluorescent rhodamine dye was filled in the wells for characterization of cross-over across wells. **g** shows the low-magnification image of dye-filled tissue (*) and no tissue (**) regions and **h** shows the high magnification image of a dye-filled region (shown in yellow box in **g**) with tissue. Well edges are seen as dark lines showing that they are above the fluid level and there is no overflow between adjacent wells. Partially filled wells indicated by a lower fluorescence were a small fraction of total wells on chip and confined to the chip boundaries as shown in Supplementary Fig. 1e (scale bar: **g** 500 µm; **h** 100 µm)

To perform the RT-LAMP reaction on a microchip from tissue samples, we developed two unique preparatory steps:

Tissue pixelation—A continuous tissue cryosection in the shape of a disc (7 µm thick) was partitioned into small tissue “pixels” and placed into the corresponding microwells. This was done by applying a centrifugal force on the tissue disc via a flexible cured polydimethylsiloxane (PDMS) block, using centrifugation in a standard centrifuge (1 min @ 1500×*g* force). When the flexible PDMS pushes its way into the wells under centrifugal forces, it shears and partitions the tissue via the sharp well edges. Supplementary Fig. 1a–d shows the images from the characterization of our chip by scanning electron microscopy (SEM), which demonstrates the sharp well edges. The tissue sticks to the well surface which was pre-silanized ((3-Aminopropyl)triethoxysilane or APTES), while the PDMS layer restores back to its original shape in the absence of force, as shown in Fig. 1d–f. Characterization of this process using SEM and fluorescence imaging for nuclei after DAPI staining is shown in Fig. 3a–f. The well edges can be clearly visualized as dark lines in the DAPI-stained fluorescent images. These data show that the tissue is completely inside the wells after the pixelation step and the tissue partitioning into pixels is complete, allowing for independent RT-LAMP reaction from the tissue in each well. The two-dimensional tissue distribution is maintained throughout the process. Supplementary Fig. 3 shows additional SEM images of tissue pixelation for a rat heart tissue section, demonstrating that the process works with other tissue types.

Bulk picoliter reagent loading—To fill the tissue-loaded wells with <175 pL of reagents per well, we developed a capillary action-based instrument-free loading technique. Five microliters of the reagent was pipetted on the chip and then the chip was immediately immersed in mineral oil. As the mineral oil has a lower density than the water-based reagents, it stays on top of the reagent-filled wells. With the mineral oil acting as an envelope, excess reagents were sheared away using air pressure while capillary forces retained the fluid only inside the wells. The process was characterized using fluorescent rhodamine dye. Figure 3g, h shows the well edges as dark lines indicating that they are above the fluid level and that there is no cross-talk between adjacent wells; 98.2% of the wells were found to be fully filled (fluorescence intensity >22 a.u.), and partially filled wells were found only near the chip borders. Supplementary Fig. 1e, f shows the complete chip data for this process. The above two processes ensure independent picoliter RT-LAMP reactions in each well starting from a tissue sample.

### Real-time microchip RT-LAMP

**Fig. 4:**
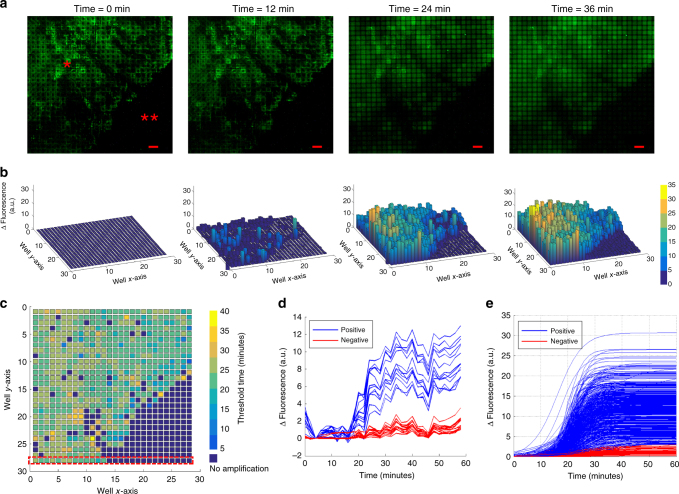
On-chip RT-LAMP for TOP2A mRNA. **a** Raw fluorescence images of real-time RT-LAMP with tissue on chip at four different time points (*Tissue, **No tissue) (scale bar: 200 µm). **b** Fluorescence bar graphs of the raw images showing a differential increase in fluorescence over time. The gain in fluorescence over time is calculated taking time = 0 image (initial) as the reference. **c** Spatial threshold analysis showing the spatially mapped threshold times. Note that the tissue boundaries are maintained throughout the reaction. Each pixel is 100 µm × 100 µm. **d** Raw amplification curves of a row marked in red in “**c**” showing positive and negative wells. **e** Fluorescence curves for all wells after curve fitting

Before performing the real-time microchip RT-LAMP reaction, the pixelated tissue was fixed using acetone for 10 min at room temperature to prevent RNA degradation^21^. Following this, the tissue was treated with proteinase K (7.5 µg mL^−1^ for 30 min), which digested the cell membrane proteins making the cells permeable to polymerase and reverse transcriptase enzymes^3^. This allowed RT-LAMP reagents to penetrate the cells and carry out amplification. As opposed to lysing the whole tissue in which scenario the tissue debris would have been completely mixed with the overlying solution in wells, using proteinase K digestion to expose the target analyte inside the tissue prevents the tissue contaminants from inhibiting RT-LAMP reaction. The amplification reagents were loaded using the previously described technique (bulk picoliter reagent loading) and the amplification was performed on a hot plate at 65 °C. Imaging was done every 2 min using a 5× objective and TRITC filter in an Olympus BX51 fluorescence microscope. The on-chip amplification reaction was completed in 36 min and the progressive product accumulation in each well was visualized as a proportional increase in the fluorescence in the corresponding well. The real-time fluorescence curves were used to calculate the threshold times for each well. Figure 4a–c shows the fluorescence images at different time points, the differential spatial fluorescence bar graphs, and the spatial threshold times, respectively. Notably, fluorescence data at the tissue margins were reliable amplification signals indicating robust measurements even at the tissue boundaries. Figure 4d shows the raw fluorescence data over time for a row of wells. A sigmoidal curve fitting was performed on the raw fluorescence curves from all the imaged wells and threshold time was calculated as shown in Supplementary Fig. 4e, f. Fluorescence curves from all the imaged wells are shown in Fig. 4e after the fitting analysis. Analyzing regions close to the tissue boundary showed that the tissue boundary was maintained during the reaction suggesting that there was no cross-talk between adjacent wells and our rapid tissue pixelation and reagent loading technique indeed isolated each well (Supplementary Fig. 4a–d). The regions without any tissue showed no non-specific amplification. To demonstrate the scalability of our technology, we also performed similar on-chip reactions from tissue on two different well sizes of 300 and 500 µm. The results are shown in Supplementary Figs. 5 and 6. To further ensure that the signal observed was not due to spurious amplification, two negative controls, one with no primers in the reaction mix and the other with RNase A (100 µg/mL for 1 h) treated tissue, were performed and no amplification was observed for either (Supplementary Fig. 7).

**Fig. 5:**
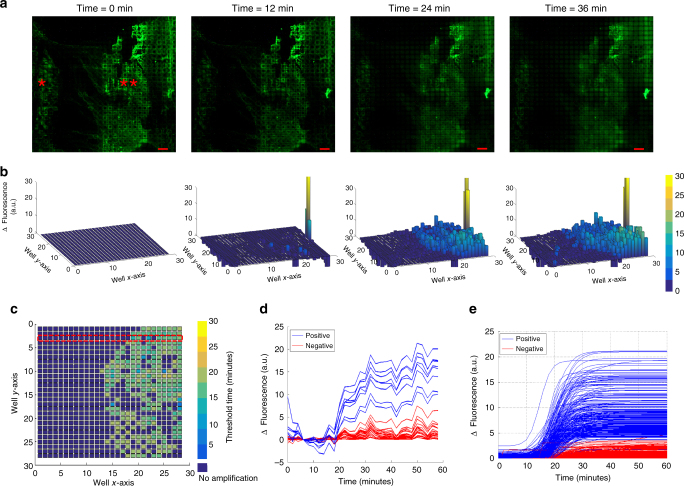
On-chip RT-LAMP: cancer vs. non-cancer control. **a** Raw fluorescence images of real-time RT-LAMP with prostate cancer tissue on right and non-cancer (mouse skeletal muscle) tissue on left of chip at four different time points (*Non-cancer, **Cancer) (scale bar: 200 µm). **b** Fluorescence bar graphs of the raw images showing a differential increase in fluorescence over time. The gain in fluorescence over time is calculated taking time = 0 image (initial) as the reference. Note the amplification occurs only for the cancerous tissue. **c** Spatial threshold analysis showing the spatially mapped threshold times. Each pixel is 100 µm × 100 µm. **d** Raw amplification curves of a row showing positive and negative wells marked in red in “**c**”. **e** Fluorescence curves for all wells after curve fitting

As a final specificity test of the on-chip assay, we loaded cancer and non-cancer (mouse skeletal muscle tissue) tissue on the same chip and performed the RT-LAMP reaction. Figure 5a, c shows the fluorescence images at different time points, the differential spatial fluorescence bar graphs, and the spatial threshold time analysis, respectively. Figure 5d, e shows the raw fluorescence data for a row of wells and the amplification plots after curve fitting analysis, respectively. Only the cancerous tissue amplified validating the specificity of our assay.

### Fourier transform infrared imaging (FTIR) control

To validate RT-LAMP based on the expression of TOP2A in different cell types (whether cancer epithelium or stroma), we performed label-free FTIR imaging on the same section prior to amplification. In this setting, only cancer epithelium are expected to express TOP2A. FTIR provides a tissue-level view of the sample without the use of dyes or other reagents that are known to degrade RNA^22, 23^. Furthermore, this technique has been previously used to provide label-free histology of prostate tissue with over 98% accuracy in determining cell types^24–26^. While IR imaging is usually performed on specialized substrates, here we made a small modification to make it compatible with our silicon microchips. As shown in Supplementary Fig. 8, a pixelated and fixed prostate cancer tissue section was imaged by FTIR spectroscopy before performing the RT-LAMP. Supplementary Fig. 9 shows the process flow with FTIR control on the same section. Supplementary Fig. 10a, b shows the bright-field image of the entire chip with tissue and the IR absorbance-based classification of the tissue section into cancerous or epithelial (red) and non-cancerous or stromal (blue) regions. Supplementary Fig. 10c–g shows the end-point fluorescence image at the 36th minute for RT-LAMP on the same chip, differential spatial fluorescence bar graphs, spatial threshold times, raw fluorescence curves for a column, and all fluorescence curves for this experiment, respectively. The results clearly demonstrate that only cancerous regions amplified. To visualize the spatial heterogeneity in TOP2A mRNA across the tissue, we plotted the fraction of cancerous tissue per well (color bar) against spatially mapped threshold times to create a 4-dimensional plot (Supplementary Fig. 10). Taking a given fraction of cancerous tissue per well as a reference for the starting sample amount in wells, the heterogeneity in TOP2A expression is evident as variation in threshold times in those wells as seen in Supplementary Fig. 10i. It can be observed that wells with none to negligible fraction of cancerous tissue (dark red) either do not amplify or have very high threshold times, whereas regions with high fraction of cancerous tissue (yellow) tend to show lower threshold times. Supplementary Fig. 11 shows a repeat experiment with FTIR.

### Comparison with mRNA FISH

**Fig. 6:**
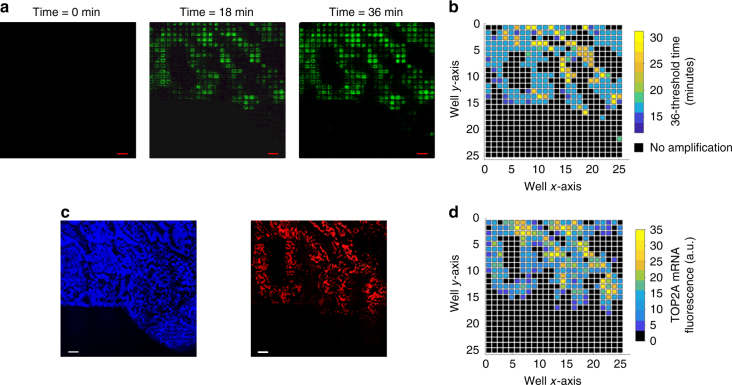
On-chip RT-LAMP with mRNA FISH on serial sections. **a** Baseline-subtracted fluorescence images of real-time RT-LAMP with tissue on chip at three different time points showing the increase in fluorescence over time (scale bar: 200 µm). **b** Spatial threshold analysis showing the spatially mapped threshold times. No amplification was observed till 36 min. **c** DAPI (blue) and TOP2A mRNA FISH (red) images of the consecutive section showing spatial heterogeneity in TOP2A mRNA expression (scale bar: 200 µm). **d** Pixelated intensity map of mRNA FISH fluorescence. The spatial pattern of TOP2A expression is similar between the two assay types. For **b**, **d**, each pixel is 100 µm × 100 µm

For further validation, we performed mRNA FISH and microchip spatial amplification on adjacent tissue sections. FISH probes for human TOP2A mRNA were confirmed to specifically stain human prostate cancer cells (PC3, LNCaP) and not stain mouse fibroblast or muscle cell lines (3T3, RAW 264.7) which do not express TOP2A (Supplementary Fig. 12). The probe sequences are provided in Supplementary Table 2. Figure 6a shows baseline-subtracted fluorescence images for the microchip amplification at three different time points, demonstrating an increase in fluorescence over time and Fig. 6b shows the spatial threshold time analysis, reflecting variations in TOP2A mRNA across the tissue. Figure 6c shows confocal fluorescence micrographs of nuclei (blue) and TOP2A mRNA FISH (red). The TOP2A mRNA FISH micrograph was then pixelated at the same spatial resolution as the spatial threshold time maps and displayed in Fig. 6d. Supplementary Fig. 13 shows an independent repeat experiment similar to the above. At 36-min reaction times, the detection sensitivity of the RT-LAMP was compatible with mFISH (Supplementary Fig. 14). However, our spatial RT-LAMP had a distinct advantage over mFISH in that it provided quantitative expression values spanning over four orders of magnitude in only 2 h (including the amplification time). This turnaround time compared very favorably against the over 2 days required to perform the mFISH experiments, and that is without taking into account the considerable time that was required to capture and process mFISH images. These important advantages make microchip amplification a suitable technique for a wide range of research and clinical applications.

### Comparison with immunohistochemistry (IHC) for TOP2A protein

As another validation, we compared the TOP2A mRNA expression by RT-LAMP and protein expression by immunostaining (IHC) on adjacent tissue sections. Supplementary Fig. 15 shows the threshold time maps with the IHC images and their overlap analysis. As can be seen in the Figure, the spatial patterns for the TOP2A mRNA and protein expression are highly concordant.

## Discussion

The technique presented here can be tuned to perform quantitative spatially mapped nucleic acid analysis of any tissue sample type on a simple hot plate and a fluorescence reader. It can also be integrated into a completely portable setup using a smartphone and an in-built heater making the technique accessible even to labs without a microscope^12^. Our technique allows analysis of small-to-large tissue regions without any cross-talk between individual tissue pixels. The tissue pixelation and bulk picoliter reagent loading were easy-to-perform steps that take only 1–2 min. This is important considering that commercial solutions for spotting arrays of nanodroplets cannot spot picoliters of volume in close spacing, have large dead volumes, and suffer from long sample loading times (serial loading of 5000 wells would take several hours)^27, 28^. Our technique can be scaled to fill larger arrays with millions of wells using the same principle in a matter of minutes. Both, the size of our chip and the spatial dimension of our wells can be tuned to meet sample size and spatial resolution requirements and we demonstrate this by showing on-chip amplification for 300 and 500 µm wells apart from the 100 µm wells. We expect the same should be possible for smaller well sizes, in which case we would make the wells deeper in order to keep similar reaction volume and kinetics as in the 100 µm wells. Our technique, which can be easily performed in routine practice, has many important clinical and biological applications such as understanding tumor heterogeneity, predicting patient outcomes, and post-operative characterization of surgical margins^29^. As such, we predict that microchip amplification will find a wide range of applications in clinical and research settings.

## Methods

### Cells and xenografts

LNCaP prostate cancer cells were obtained from and verified by the American Type Culture Collection (ATCC). The cells were cultured per the recommended standard in a 37 °C humidified incubator with 5% CO_2_ atmosphere.

LNCaP subcutaneous xenografts were grown in immunocompromised nude mice (Jackson Laboratory). LNCaP cells were first incubated and grown to confluence. Then, these confluent cells were suspended in 10% Matrigel at a concentration of 2 × 10^7^ cells/60 μL and 60 μL of this Matrigel–cell solution was injected subcutaneously into both flanks of the animal. The mice were then monitored daily for the presence of tumors. As soon as tumors were visible, volume measurements were taken using digital calipers twice a week, and tumor volumes were calculated using the formula volume = (length × width^2^)/2. Once tumors reached 50,000 mm^3^ in volume, the mice were sacrificed by overdosing with isofluorane anesthesia, after which the tumors were immediately excised and divided to be placed in optimal cutting temperature compound (OCT), or 4% PFA. All animal experiments were performed according to the guiding principles of the American Physiologic Society and approved by the Institutional Animal Care and Use Committee (IACUC protocol #17106—Dobrucki, PI) at the University of Illinois at Urbana-Champaign.

### Primer sequences

All primer sequences for the RT-LAMP and RT-PCR reactions were synthesized by Integrated DNA Technology (IDT) and are listed in Supplementary Table 1. Primerexplorerv4 (https://primerexplorer.jp/e/) was used for designing the RT-LAMP primers for TOP2A mRNA. The cDNA sequence for TOP2A was obtained from NCBI database using NCBI Reference Sequence: NM_001067.3. https://www.ncbi.nlm.nih.gov/nuccore/NM_001067.3.

### Off-chip reactions

RT-LAMP assay was designed to target the TOP2A mRNA. The RT-LAMP assay comprised of the following components: 1× final concentration of the isothermal amplification buffer (New England Biolabs), 1.4 mmol L^−1^ each of deoxy-ribonucleoside triphosphates (dNTPs), 10 mmol L^−1^ of MgSO4 (New England Biolabs), and 0.4 mol L^−1^ of Betaine (Sigma-Aldrich). These components were prepared in bulk and stored at −20 °C between experiments. In addition to the buffer components, 1 μL of primer mix consisting of 0.2 μM of F3 and B3, 1.6 μM FIP and BIP, and 0.8 μM of LoopF and LoopB, 0.64 U μL^−1^ Bst 2.0 WarmStart DNA Polymerase (New England Biolabs), 0.08 U μL^−1^ AMV reverse transcriptase (New England Biolabs), and 1× EvaGreen (Biotium), a double-stranded DNA (dsDNA) intercalating dye, was included in the reaction. Ten microliter template of the appropriate concentration and 1.92 μL of DEPC-treated water (Invitrogen) were added to make the final reaction volume 25 μL.

The RT-PCR reaction was carried out using the RNA UltraSense ™ One-Step Quantitative RT-PCR System (Thermofisher) according the manufacturer’s instructions. A 50-μL reaction mix contained 2.5 µL of RNA UltraSense™ Enzyme Mix, 10 µL of RNA UltraSense™ 5X Reaction Mix™, 0.5 µL of 10 µM forward primer (100 nM reaction concentration), 0.5 µL of 10 µM backward primer (100 nM reaction concentration), 1 µL of SYBR Green dye (Thermofisher), and 34.5 µL of template of the appropriate starting concentration.

Template for the RT-LAMP reactions included either RNA extracted from LNCaP cells or LNCaP prostate xenograft tissues, or whole LNCaP cells spiked in the reaction mix. All RNA extractions were performed using the RNeasy Mini Kit (Qiagen) according to the manufacturer’s instructions. All RT-LAMP and RT-PCR reactions consisted of non-template negative controls, the amplification of which indicated a contaminated test.

All the off-chip LAMP tests were carried out in 0.2 mL PCR reaction tubes in an Eppendorf Mastercycler® realplex Real-Time PCR System. The tubes were incubated at 60 °C for 60 min in the thermocycler, and fluorescent data were recorded every 1 min. The off-chip PCR tests were conducted on the same thermocycler but with the following protocol: RT incubation at 50 °C for 50 min, 2 min DNA denaturation at 95 °C, and 50 cycles of thermocycling from 95 °C (15 s) to 60 °C (30 s). Fluorescence data were recorded after each cycle of the reaction. Triplicates were done for each reaction.

### Chip fabrication and chip silanization

Undoped silicon wafers (University Wafers) were piranha cleaned for 10 min and a 160 nm of silicon oxide was thermally grown in a furnace at 1150 °C for 90 min. A 2-μm layer of positive photoresist AZ1518 (AZ Electronic Materials) was spin-coated on the unpolished side of the wafer and was soft-baked on a hot plate at 110 °C for 8 min. The same process was repeated on the polished size of the wafer. The photoresist on the shiny side was patterned using an EVG 620 aligner with a high-resolution transparency mask (FineLine Imaging). The wafer was then developed in AZ400K (AZ Electronics) to remove the exposed regions for 1 min. The unprotected silicon oxide was etched in 10:1 buffered oxide etchant (VWR) to reveal the underlying bare silicon. This was followed by stripping the wafer of photoresist in a Remover PG (MicroChem) at 70 °C for 30 min. The wafer was then anisotropically etched in a TMAH bath (1:1 TMAH:DI) for 100 min at 80 °C to etch inverted square pyramidal wells with sharp well edges. To passivate the chip, 125 nm of silicon oxide was thermally grown in a furnace at 1150 °C for 90 min.

To render a positive charge on well surfaces for tissue adhesion, silicon chips were silanized with functional groups using APTES. The chips were dipped in a glass jar containing 0.2% APTES for 60 s. The chips were then dipped five times in a separate vessel containing distilled water. This step was repeated three more times with the water being replaced between each step. The silanized chips were stored in a desiccator, and were used within 15 days of silanization.

### Tissue fixation and proteinase K digestion

The frozen tissue was cryo-sectioned at a thickness of 7 µm onto the center of the microchip and stored at −20 °C. Once ready for use, the tissue was taken out of the −20 °C freezer and dried immediately to minimize RNase activity within the tissue. A short heating step at 105 °C for 5 s was incorporated to ensure that the tissue is stabilized on the chip. A clean block of PDMS was then placed on top of the tissue and the whole PDMS–chip conjugate was centrifuged at 3000 rpm for 1 min to press the tissue into the wells in a process known as tissue pixelation. The block of PDMS was discarded, and a second heating step at 105 °C for 7 s was carried out to ensure the tissue is firmly adhered and stable on the chip.

A standard acetone fixation protocol was followed to fix the tissue onto the chip. The chip was placed on a small glass Petri dish filled with acetone and incubated at −20 °C for 10 min. After the incubation step, the chip underwent a series of wash steps. First, half of the acetone was discarded from the petri dish and an equal amount of cold phosphate buffered saline (PBS) (Fisher Scientific) was poured into the Petri dish to replace the acetone. The Petri dish was shaken for 30 s and the step was repeated. The chip was then placed on a Petri dish containing cold PBS and shaken for 2 min. This was followed by a rinse with DEPC-treated water at room temperature for one minute. The chip was then air dried for 1 min.

The chip was placed on a Petri dish with Proteinase K at a concentration of 7.5 µg mL^−1^ and incubated at 45 °C for 30 min. Once the digestion was complete, the proteinase K was denatured by heating the chip at 95 °C for 90 s. The chip was washed with PBS for 10 s and DEPC-treated water for 30 s to remove the residual proteinase K.

### Bulk loading and on-chip RT-LAMP reactions

A 25-µL reaction mix was prepared for a single on-chip test, and 5 µL of the reaction mix was pipetted onto the tissue/wells, and immediately coated with a layer of mineral oil. The chip was then placed in a Petri dish covered with mineral oil and degassed for 5 min to remove any air bubbles. After degassing, the chip was dipped in mineral oil, and an air pressure is applied at an angle to shear off and remove excess reagents from top of the wells. The chip was then placed on a copper bowl and placed on a hotplate under a fluorescent microscope to perform the on-chip RT-LAMP reactions.

The on-chip reactions were carried on a commercial hotplate at 65 °C for 60 min and imaged every 2 min under an Olympus BX51 fluorescence microscope with 3.6 s exposure settings and 16× gain. TRITC filter was used for imaging the EvaGreen fluorescent dye.

### Amplification data analysis

The off-chip amplification and standard curves were plotted using a MATLAB script. The threshold time for each curve was taken as the time required for each curve to reach 20% of the maximum intensity. For on-chip reactions, the raw fluorescent intensity on-chip was extracted from each well and was plotted against time to generate the raw fluorescence curves. Each raw amplification curve was fitted to a sigmoidal curve using a four-point parameter modeling (Supplementary Fig. 6). The following equation was used for the analysis:

$$f = y_0 + \frac{a}{{1 + e^{ - \left( {\frac{{x - x_0}}{b}} \right)}}}$$

where *f* = fluorescence intensity, *y*_0_ = background fluorescence at time = 0 min, *a* = difference between the initial and final fluorescent intensity, *x* = time point of analysis, *x*_0_ = inflection point of the curve, and *b* = slope of the curve. The positive and negative wells were differentiated on the basis of the *R*^2^ value of the sigmoidal fit and the parameters *a* and *x*_0_. The threshold time was taken as the point of inflection. Negative wells had a combination of low *R*^2^ value, low *a* value, or a very high threshold time (*x*_0_ > 50 min).

### FTIR spectroscopic imaging

IR images were acquired using a Spotlight 400 (Perkin Elmer) IR imaging instrument at a spatial pixel size of 6.25 µm × 6.25 µm and a spectral resolution of 4 cm^−1^ at an under-sampling ratio of 2. The spectral profile of a pixel was truncated to a spectral range of 4000–720 cm^−1^ for ease of data handling and classification. The sample was imaged in the reflection–absorption mode. Data were water vapor corrected and used for classification using the previously described Bayesian approach, metrics, and algorithms for prostate tissue. Further details of FTIR spectroscopy is provided in Supplementary Note 1.

### TOP2A mRNA FISH probe design

FISH probes targeting human TOP2A mRNA (NM_001067.3, 3490 bp to 5753 bp) were designed using Stellaris® Probe Designer (version 4.2, LGC Biosearch Technologies, USA). Probe sequences with 85% or greater homology with mouse TOP2A mRNA complementary sequences were excluded. The resulting set of 36 mRNA FISH probes with 3′ Quasar 670 dye label was synthesized by LGC Biosearch Technologies. Probe sequences are listed in Supplementary Table 2.

### Validation of TOP2A mRNA FISH on cell lines

Probe specificity was validated on TOP2A-positive human cell lines (LNCaP and PC-3, generous gifts from Dr. Stephen J. Murphy, Mayo Clinic) and mouse cell lines as negative controls (3T3 and RAW 264.7, purchased from ATCC) following previously published procedures^6^ and protocols provided by the probe supplier. Briefly, 1 × 10^5^ cells were seeded on an 18 mm round #1 coverglass in each well of a 12-well cell culture plate. After adhering, cells were washed with PBS and fixed with 4% paraformaldehyde for 10 min at room temperature. Cells were then permeabilized with 70% (v/v) ethanol for 24 h at 4 °C. Ethanol was aspirated and Wash Buffer A (LGC Biosearch Technologies) was added. After incubation for 5 min at room temperature, the coverglass was transferred face-down onto Parafilm with 100 μL of Hybridization Buffer (LGC Biosearch Technologies) containing probes. After incubation for 16 h in the dark at 37 °C in a sealed humidified chamber, the coverglass was washed with Wash Buffer A in the dark at 37 °C for 30 min. Nuclei were counterstained with Hoechst 33342 (Thermofisher, USA) for 30 min. The coverglass was then washed with Wash Buffer B (LGC Biosearch Technologies) for 5 min before mounting on slides containing Vectashield Mounting Medium, and sealed using nail polish. FISH probes and nuclei were imaged using a Leica SP8 UV/Visible Laser Confocal Microscope (Leica, Germany) with a 63× oil-immersion objective.

### TOP2A mRNA FISH on prostate tumor tissue

Experimental procedures for FISH staining were reported previously^30^ and provided by the probe supplier. Tumor tissue sections were stored at −80 °C and equilibrated to room temperature before use. The tissue-mounted slides were immersed in 4% paraformaldehyde fixation buffer for 10 min at room temperature and then treated in the same way as cell lines for mRNA FISH, with the exception that a 20× oil objective was used for image collection.

### mRNA FISH and microchip amplification overlap analysis

A pixelated mRNA FISH image with the same resolution as the spatial threshold map was created by generating a grid over the mRNA FISH image and taking the mean fluorescence intensity of the neighboring pixels within one unit or pixel of the grid. Since the placement of grid is done manually and is variable, we translated the grid in “*x*” and “*y*” directions to find the best overlap conditions between the two techniques. Note that the splitting of an mRNA FISH signal into pixels can artificially increase the sensitivity of FISH during the pixelation process (one fluorescent FISH signal can be split into maximum of four positive pixels depending on the grid line placement). This artifact is visible when we are shifting the grid by small distances (5 µm). (Supplementary Fig. 14). Further information on this overlap analysis is provided in Supplementary Note 2.

### IHC for TOP2A protein

The immunostaining (IHC) experiment was performed at the Mayo Pathology Research Core and followed our previously published protocol^31^ with minor modifications. Briefly, the staining procedure used Leica Bond RX Stainer (Leica, Buffalo, IL). The frozen slides were pulled from −80° and placed in a desiccator to dry overnight. The slides were fixed in 4% paraformaldehyde for 10 min and then rinsed in PBS. The slides were loaded wet onto the stainer and the epitope retrieved on-line using Epitope Retrieval 2 (EDTA; Leica, Buffalo, IL) for 10 min. The primary antibody TopoIIA (Clone 3F6, Leica Microsystems) was diluted to 1:200 in Bond Diluent (Leica) and incubated for 15 min.

The detection system used was Polymer Refine Detection System (Leica, Buffalo, IL). This system includes the hydrogen peroxidase block, secondary antibody polymer, DAB, and hematoxylin. Once completed, slides were removed from the stainer and rinsed for 5 min in tap water. Slides were dehydrated in increasing concentrations of ethyl alcohol and xylene prior to permanent coverslipping in xylene-based media.

### Data availability

The authors declare that all the data supporting the findings of this study are available within the paper and its Supplementary Information files or upon reasonable request.

## Electronic supplementary material

Supplementary Information

Peer Review File

## References

[1] Espina V (2006). Laser-capture microdissection. Nat. Protoc..

[2] Armani M, Tangrea MA, Smela E (2011). Quantifying mRNA levels across tissue sections with 2D-RT-qPCR. Anal. Bioanal. Chem..

[3] Bagasra O (2007). Protocols for the in situ PCR-amplification and detection of mRNA and DNA sequences. Nat. Protoc..

[4] Moffitt JR (2016). High-performance multiplexed fluorescence in situ hybridization in culture and tissue with matrix imprinting and clearing. Proc. Natl Acad. Sci. USA.

[5] Femino, A. M., Fay, F. S., Fogarty, K. & Singer, R. H. Visualization of single RNA transcripts in situ. *Science* **280**, 865–867 (1998). 10.1126/science.280.5363.585 9554849

[6] Raj A, van den Bogaard P, Rifkin SA, van Oudenaarden A, Tyagi S (2008). Imaging individual mRNA molecules using multiple singly labeled probes. Nat. Methods.

[7] Lyubimova A (2013). Single-molecule mRNA detection and counting in mammalian tissue. Nat. Protoc..

[8] Ståhl PL (2016). Visualization and analysis of gene expression in tissue sections by spatial transcriptomics. Science.

[9] Achim K (2015). High-throughput spatial mapping of single-cell RNA-seq data to tissue of origin. Nat. Biotechnol..

[10] Satija R, Farrell JA, Gennert D, Schier AF, Regev A (2015). Spatial reconstruction of single-cell gene expression data. Nat. Biotechnol..

[11] Morton ML (2014). Identification of mRNAs and lincRNAs associated with lung cancer progression using next-generation RNA sequencing from laser micro-dissected archival FFPE tissue specimens. Lung Cancer.

[12] Damhorst GL (2015). Smartphone-imaged HIV-1 reverse transcription loop-mediated isothermal amplification (RT-LAMP) on a chip from whole blood. Engineering.

[13] Notomi T (2000). Loop-mediated isothermal amplification of DNA. Nucleic Acids Res..

[14] Siegel RL, Miller KD, Jemal A (2015). Cancer statistics, 2015. CA Cancer J. Clin..

[15] Collins AT, Berry PA, Hyde C, Stower MJ, Maitland NJ (2005). Prospective identification of tumorigenic prostate cancer stem cells. Cancer Res..

[16] Feldman BJ, Feldman D (2001). The development of androgen-independent prostate cancer. Nat. Rev. Cancer.

[17] Pienta, K. J. & Bradley, D. Mechanisms underlying the development of androgen-independent prostate cancer. *Clin. Cancer Res*. **12**, 1665-1671 (2006). 10.1158/1078-0432.CCR-06-0067 16551847

[18] Tomlins SA (2007). Integrative molecular concept modeling of prostate cancer progression. Nat. Genet..

[19] Koivisto P (1997). Androgen receptor gene amplification: a possible molecular mechanism for androgen deprivation therapy failure in prostate cancer. Cancer Res..

[20] Cheville, J., Karnes, R. & Therneau, T. Gene panel model predictive of outcome in men at high-risk of systemic progression and death from prostate cancer after radical retropubic prostatectomy. *J. Clin. Oncology* **26**, 3930–3936 (2008). 10.1200/JCO.2007.15.6752 PMC2654311 18711181

[21] Goldsworthy SM, Stockton PS, Trempus CS, Foley JF, Maronpot RR (1999). Effects of fixation on RNA extraction and amplification from laser capture microdissected tissue. Mol. Carcinog..

[22] Wang H (2006). Histological staining methods preparatory to laser capture microdissection significantly affect the integrity of the cellular RNA. BMC Genom..

[23] Fend F (1999). Immuno-LCM: laser capture microdissection of immunostained frozen sections for mRNA analysis. Am. J. Pathol..

[24] Fernandez DC, Bhargava R, Hewitt SM, Levin IW (2005). Infrared spectroscopic imaging for histopathologic recognition. Nat. Biotechnol..

[25] Bhargava R (2007). Towards a practical Fourier transform infrared chemical imaging protocol for cancer histopathology. Anal. Bioanal. Chem..

[26] Bhargava R, Fernandez DC, Hewitt SM, Levin IW (2006). High throughput assessment of cells and tissues: Bayesian classification of spectral metrics from infrared vibrational spectroscopic imaging data. Biochim. Biophys. Acta.

[27] Super small amount fixed-quantity dispenser NANO MASTER SMP-III | Musashi engineering. Available at: http://www.musashi-engineering.co.jp.e.cn.hp.transer.com/products/100_3-1-2-2.html (Accessed 3 April 2017).

[28] NanoQuot^TM^ Microplate Dispenser. Available at: http://www.biotek.com/about/news.html?id=8672 (Accessed 23 December 2016).

[29] Itonaga M (2016). Novel methodology for rapid detection of KRAS mutation using PNA-LNA mediated loop-mediated isothermal amplification. PLoS ONE.

[30] Itzkovitz S, Lyubimova A, Blat I (2012). Single-molecule transcript counting of stem-cell markers in the mouse intestine. Nat. Cell..

[31] Karnes RJ (2010). The ability of biomarkers to predict systemic progression in men with high-risk prostate cancer treated surgically is dependent on ERG status. Cancer Res..

