# Supplementary material for: Pixelated spatial gene expression analysis from tissue
Source: Nat Commun. 2018 Jan 15;9:202. doi: 10.1038/s41467-017-02623-9 (PMC5768672; doi:10.1038/s41467-017-02623-9)
Supplement: Supplementary file 1 — Supplementary Information [file 41467_2017_2623_MOESM1_ESM.pdf]

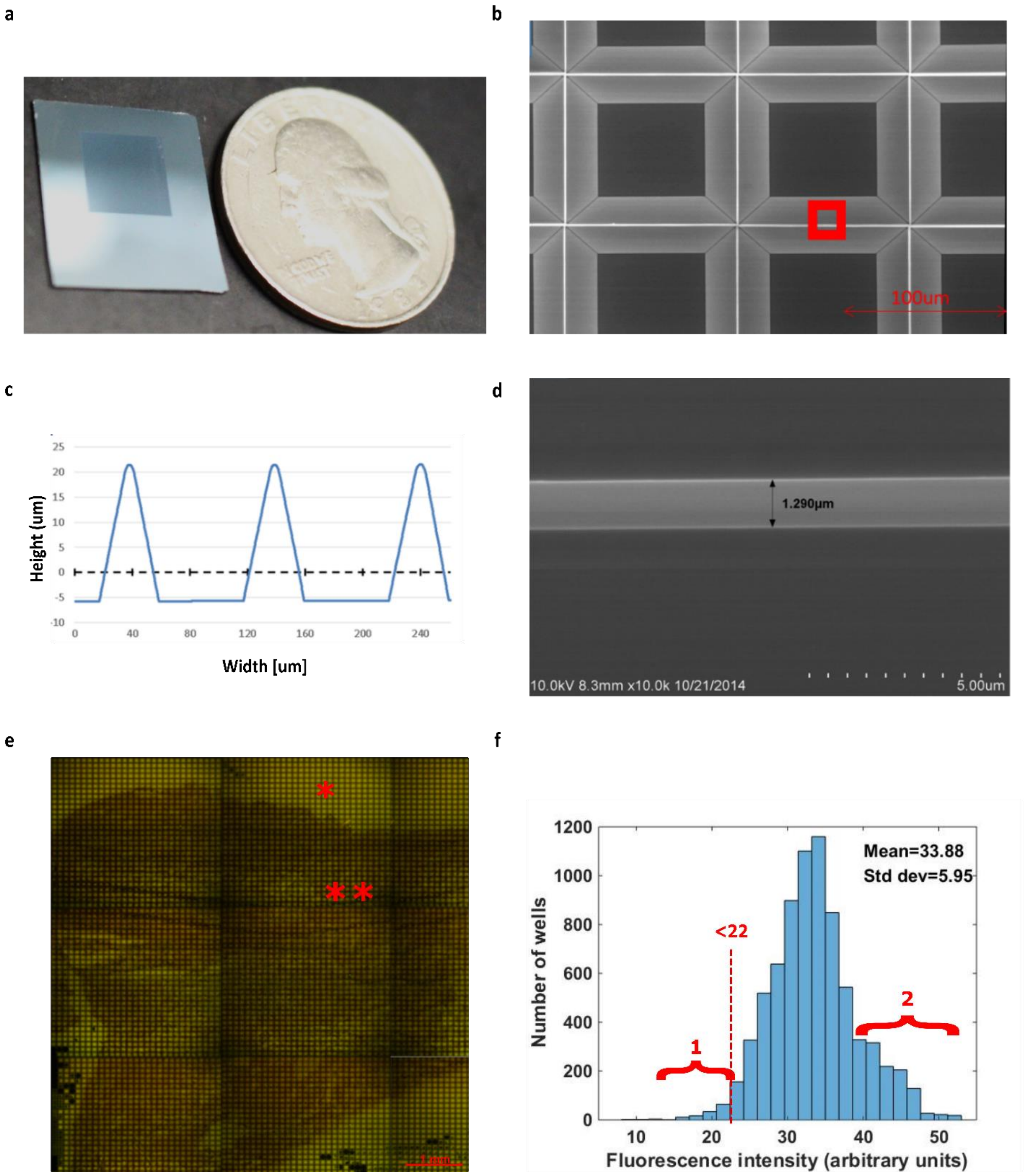

Supplementary Figure 1 | Chip and bulk loading characterization. a, Optical image of the chip beside a quarter. The dark region in the chip is the array of microwells. **b,** SEM image of the wells. **c,** Surface profilometer measurement of fabricated silicon oxide micro wells showing the depth of the wells. **d,** SEM image of the sharp well edge shown as red box in image b. The edge width is close to 1μm. **e,** Tiled fluorescent image of the complete chip showing filling distribution of the wells using Rhodamine dye (*no tissue, **tissue). It can be seen that only a few wells at the chip border are partially filled. The well edges are dark and clearly visible indicating no cross-talk between adjacent wells. **f,** Histogram showing the well fluorescence distribution after filling. Note that lower fluorescence is attributed to partially filled wells (region 1) and higher fluorescence values are for wells without tissue (region 2). The wells with fluorescence lower than 22 units were found to be partially filled and were present only around the chip borders.

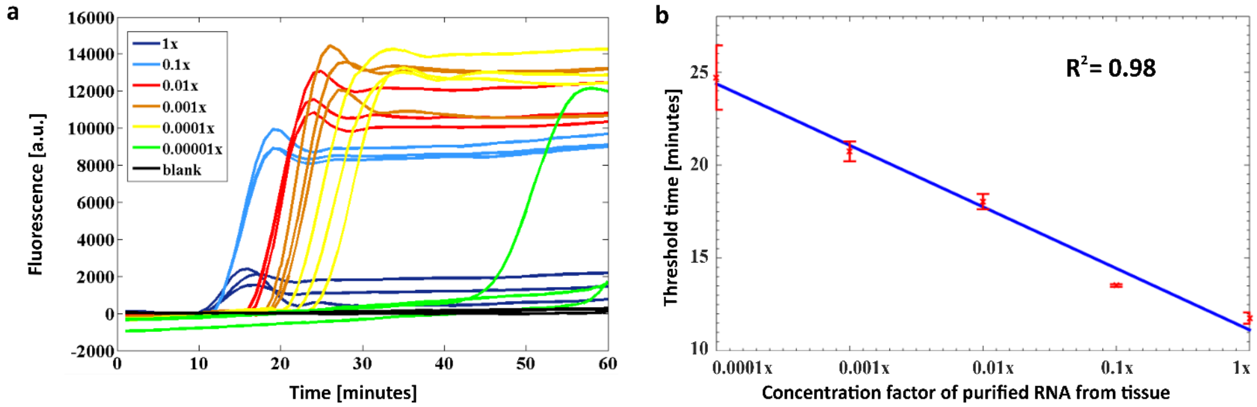

Supplementary Figure 2 | RT-LAMP assay with purified RNA from tissue xenograft. Amplification curves (a) and standard curve (b) of the TOP2A mRNA RT-LAMP with purified total RNA extracted from tissue. 1x=2450ng (7um thick cryosection of PCa xenograft) of total RNA from purified RNA from tissue per reaction. All the reactions had 3 replicates (n=3) and the error bars show the standard deviation. (s.d)

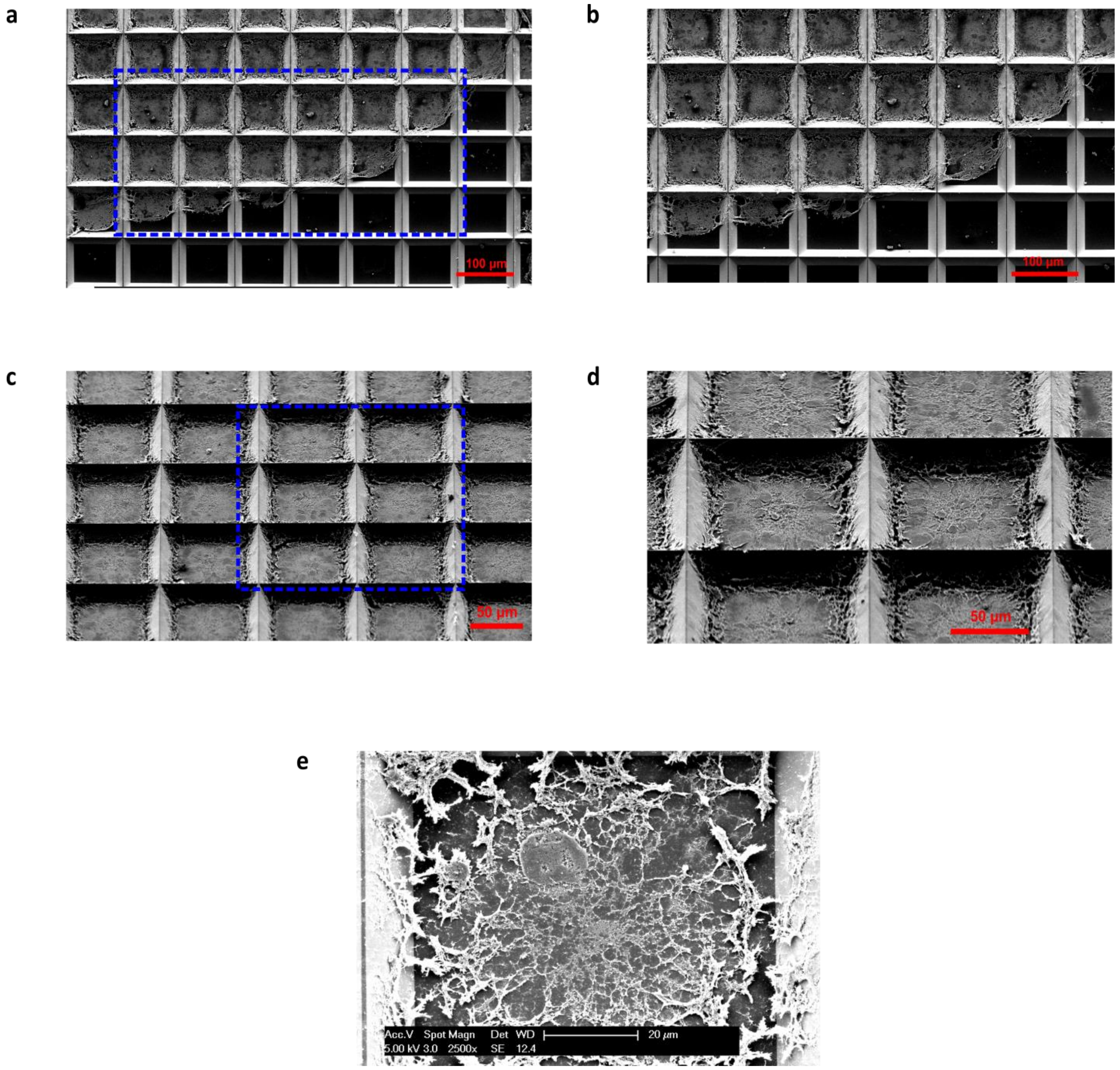

Supplementary Figure 3 | SEM characterization of rat heart tissue pixelation. a-e, Tissue partitioning and division into small pixels can be clearly visualized as tissue inside the wells. The blue box in Supplementary Fig. 3a is shown in Supplementary Fig. 3b and the blue box in Supplementary Fig. 3c is shown in Supplementary Fig. 3d. Supplementary Fig. 3e shows pixelated tissue inside a single well.

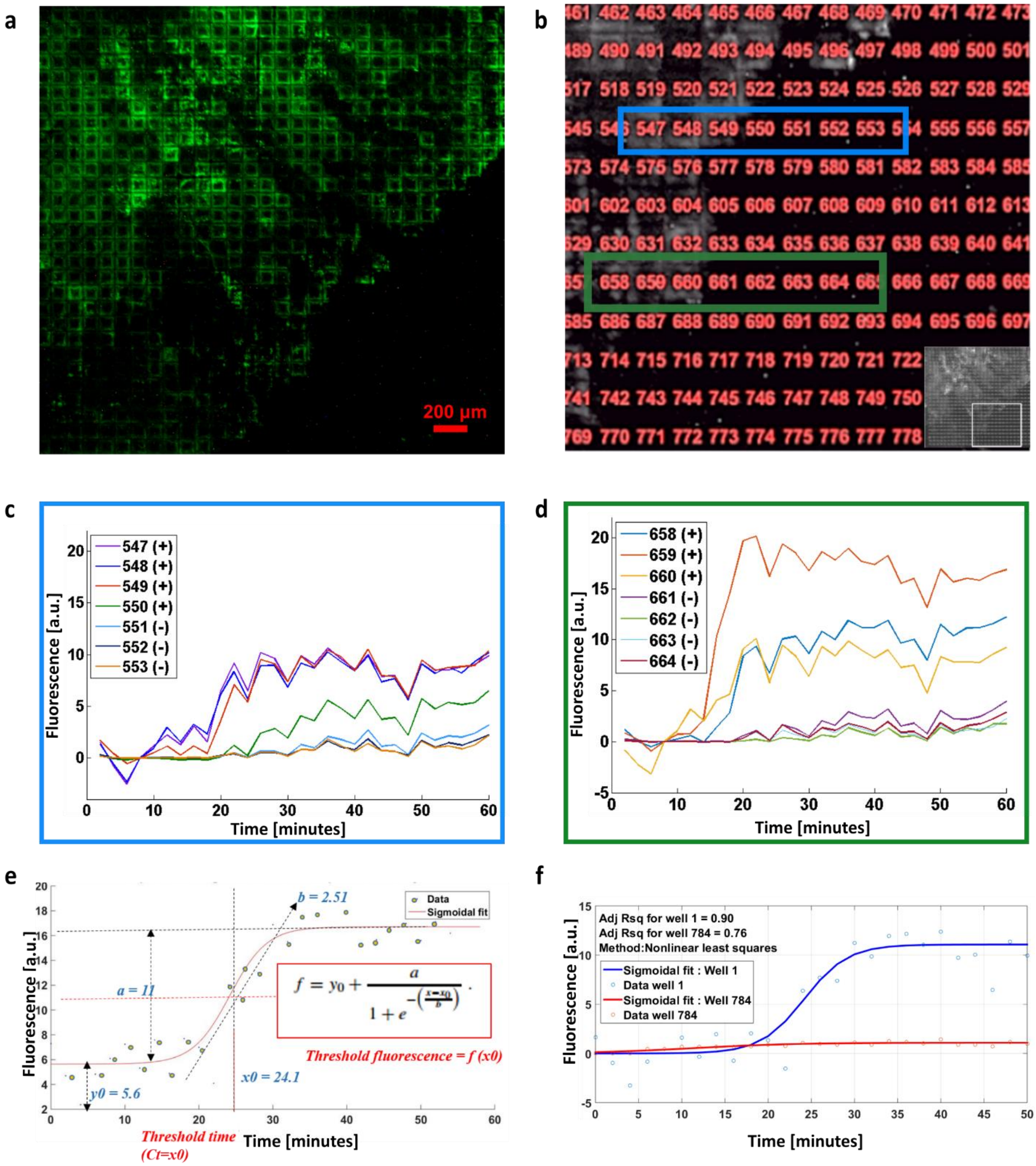

$$f = y_0 + \frac{a}{1 + e^{-\left(\frac{x - x_0}{b}\right)}}$$

Supplementary Figure 4 | Regional Image Analysis for Figure 3 in the main paper. a, Raw fluorescence image at time = 0 showing regions with and without tissue. **b,** Zoomed in processed image with numbered wells. The inset shows the entire processed image. **c-d,** Raw amplification curves for marked regions (blue and green) showing that the positive wells (with tissue) amplify while the adjacent negative wells do not. The tissue boundary remains preserved during the amplification reaction confirming that there is no cross talk between adjacent wells. **e,** 4 point parameter model used for the sigmoidal fitting of the raw amplification curves. The equation for the sigmoidal fit is given in the red box inside the figure and the corresponding parameters are represented in the data fit shown as an example. The threshold time was taken as the point of inflexion of the amplification curve. **f,** Representative amplification curve with sigmoidal fit from a positive and negative well**.**

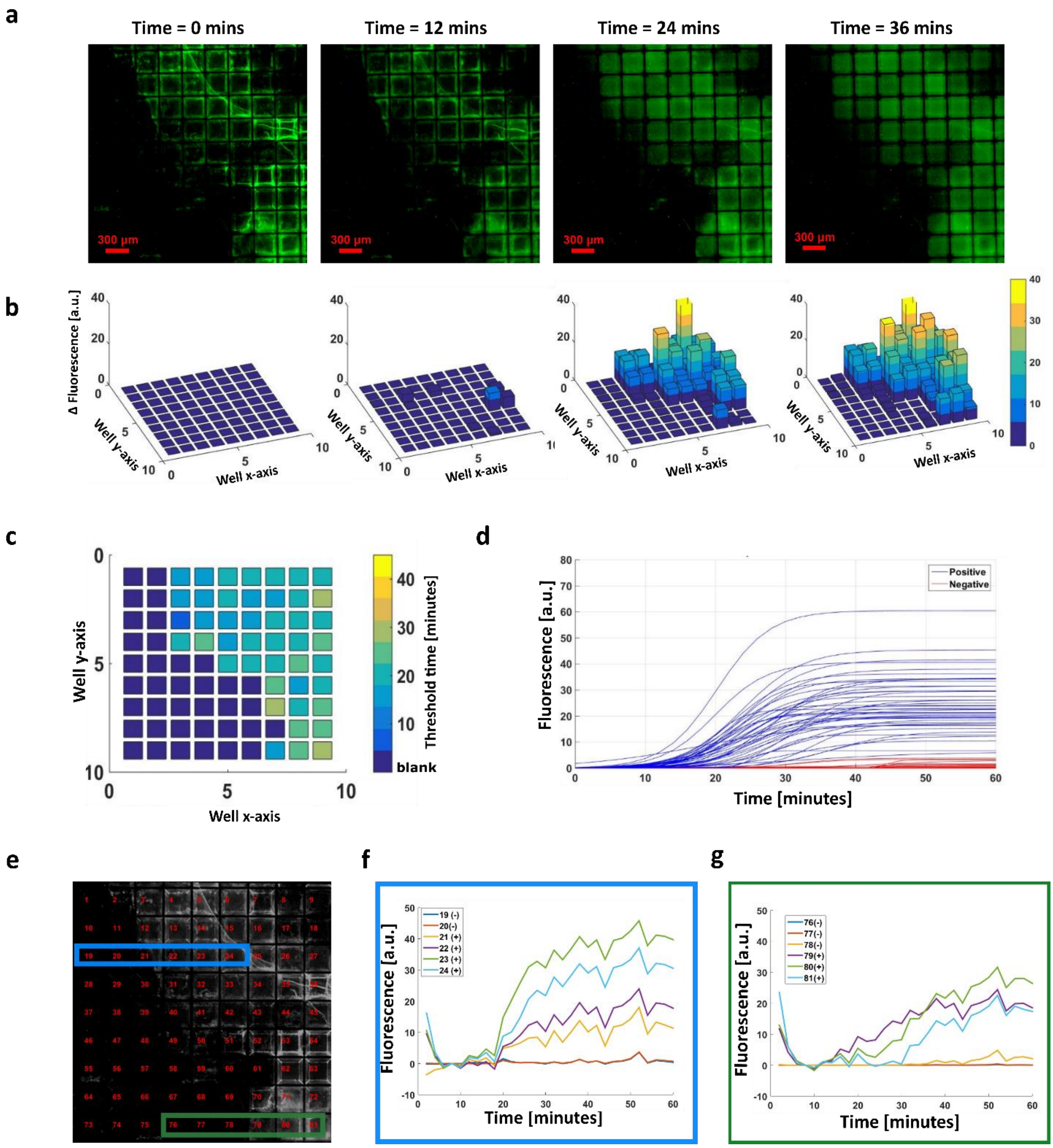

Supplementary Figure 5 | On-chip RT-LAMP with 300 µm well size. a, Raw fluorescence images of real-time RT-LAMP with tissue on chip at four different time points. **b,** Corresponding 3D fluorescent bar graphs of the raw images showing a differential increase in fluorescence over time. The gain in fluorescence over time is calculated taking time= 0 image (initial) as the reference. **c,** Spatial threshold analysis showing the spatially mapped threshold times. Note that the tissue boundaries are maintained during reaction. Threshold time=0 refers to blanks. **d,** Amplification curves for all wells after curve fitting. **e,** Processed image with numbered wells. **f-g,** Raw amplification curves of the regions marked in blue and green in "e" showing the differences in fluorescence between tissue and no tissue regions over time.

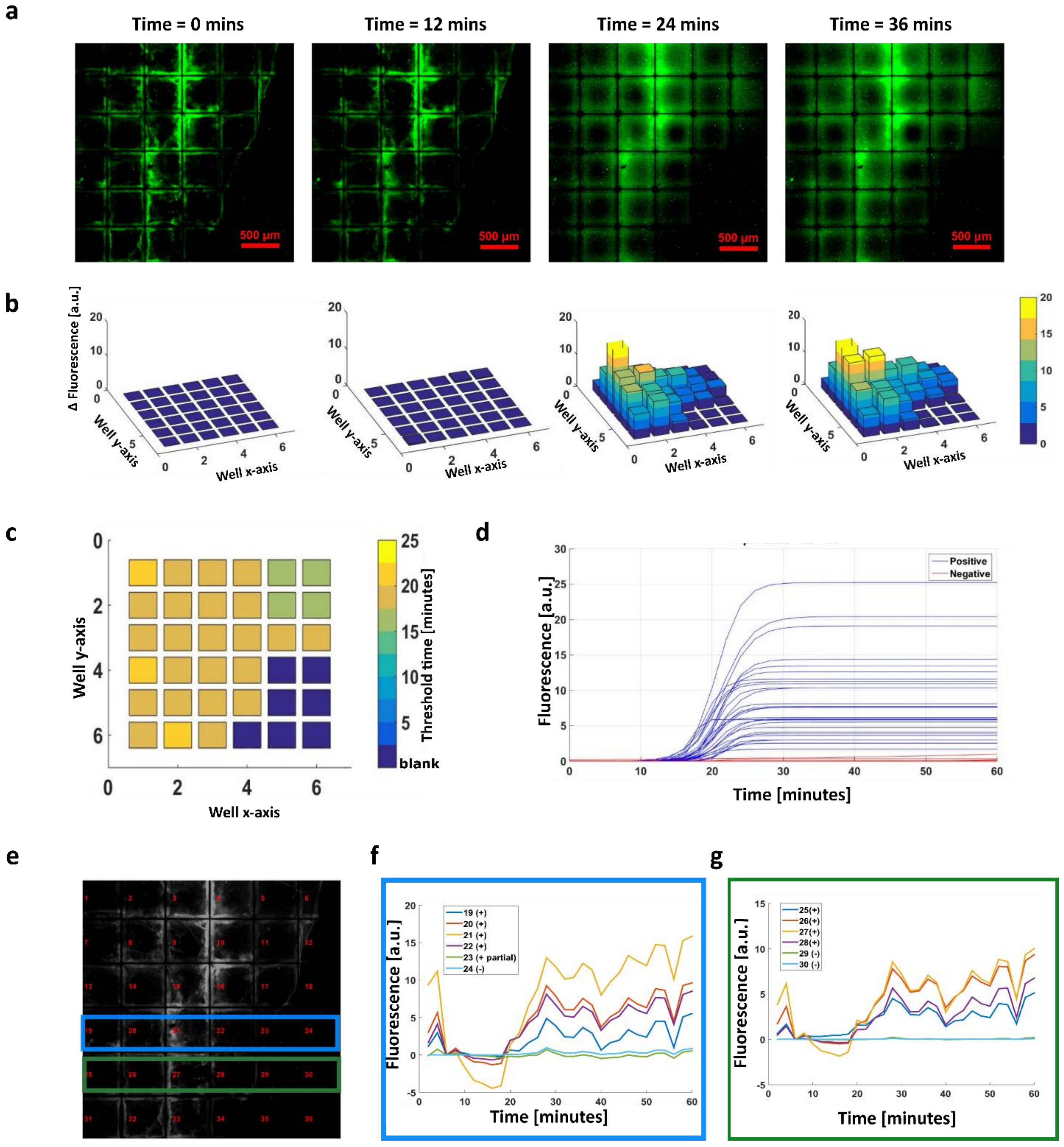

Supplementary Figure 6 | On-chip RT-LAMP with 500 µm well size. a, Raw fluorescence images of real-time RT-LAMP with tissue on chip at four different time points. **b,** Corresponding 3D fluorescent bar graphs of the raw images showing a differential increase in fluorescence over time. The gain in fluorescence over time is calculated taking time= 0 image (initial) as the reference. **c,** Spatial threshold analysis showing the spatially mapped threshold times. Note that the tissue boundaries are maintained during reaction. Threshold time=0 refers to blanks. **d,** Amplification curves for all wells after curve fitting. **e,** Processed image with numbered wells. **f-g,** Raw amplification curves of the regions marked in blue and green in "e" showing the differences in fluorescence between tissue and no tissue regions over time.

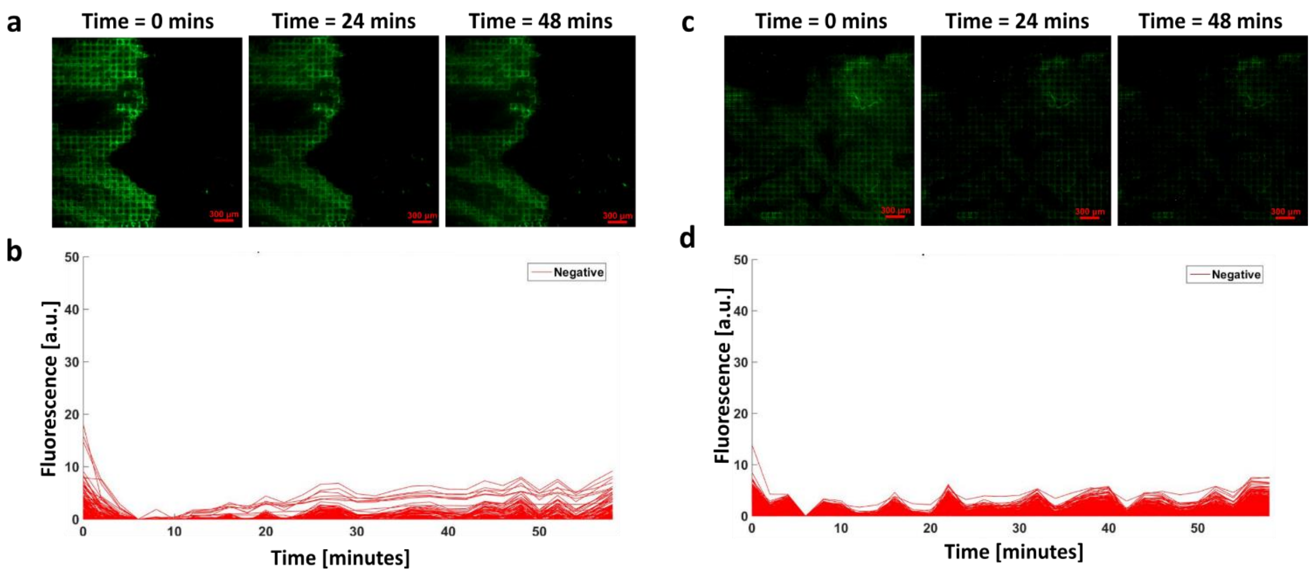

Supplementary Figure 7 | On-chip RT-LAMP negative controls. a-b, Raw fluorescence images of real-time RT-LAMP reaction with no primers added at three different time points and the corresponding amplification curves from all wells showing no amplification during the entire reaction period. **c-d,** Raw fluorescence images of real-time RT-LAMP reaction with RNase treated tissue at three different time points and the corresponding amplification curves from all wells showing no amplification during the entire reaction period.

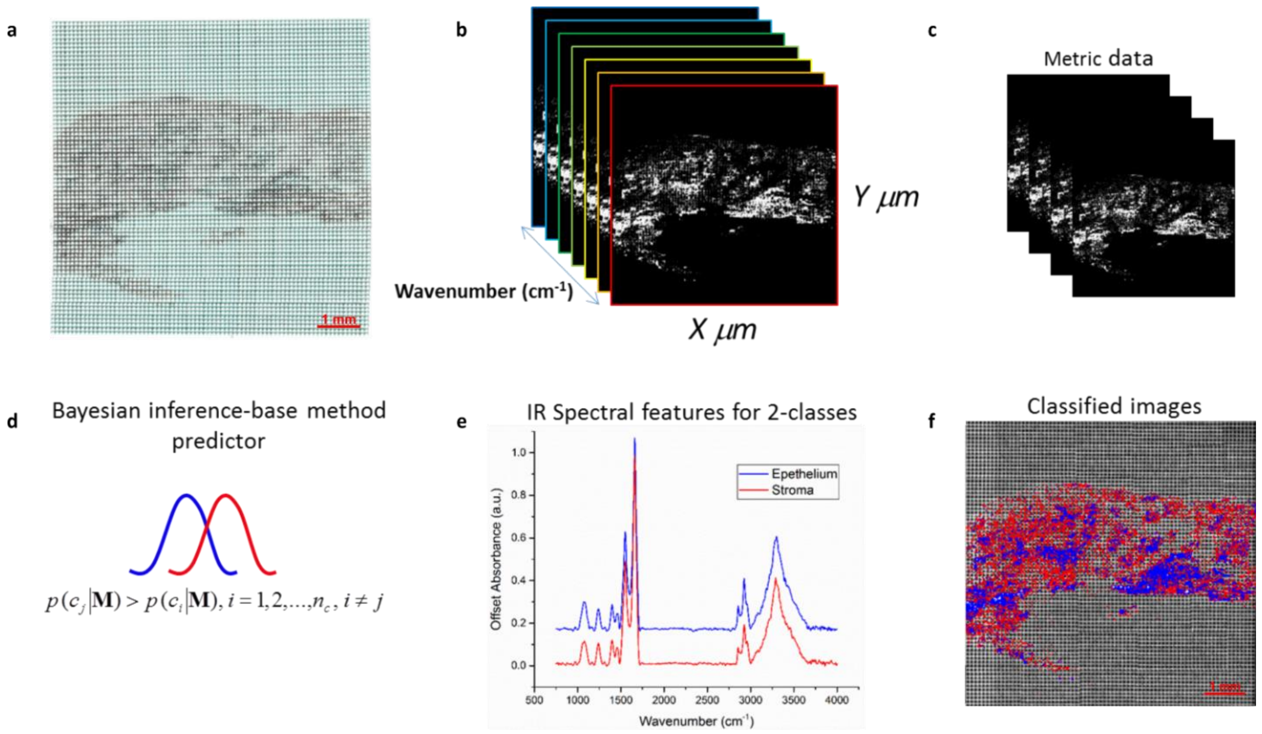

Supplementary Figure 8 | FT-IR spectral signature characterization. (a) Bright-field image of unstained tissue sample in which it is difficult to discern tumor from stroma. (b) IR imaging provides both spatial (x-y) and spectral data. (c) The data are reduced to biologically relevant metrics that characterize differences in cell types and can be used to recognize tissue histology without dyes or stains. (d) Absorbance distributions of different cell types can be separated using machine learning methods (here a modified Bayesian approach). (e) Spectral characteristics of epithelial and stromal cells, showing subtle differences that can be directly detected by spectral changes. (f) Cellular type identified by machine learning using IR spectral data. The red color indicates epithelial tumor cells and blue color indicates stroma.

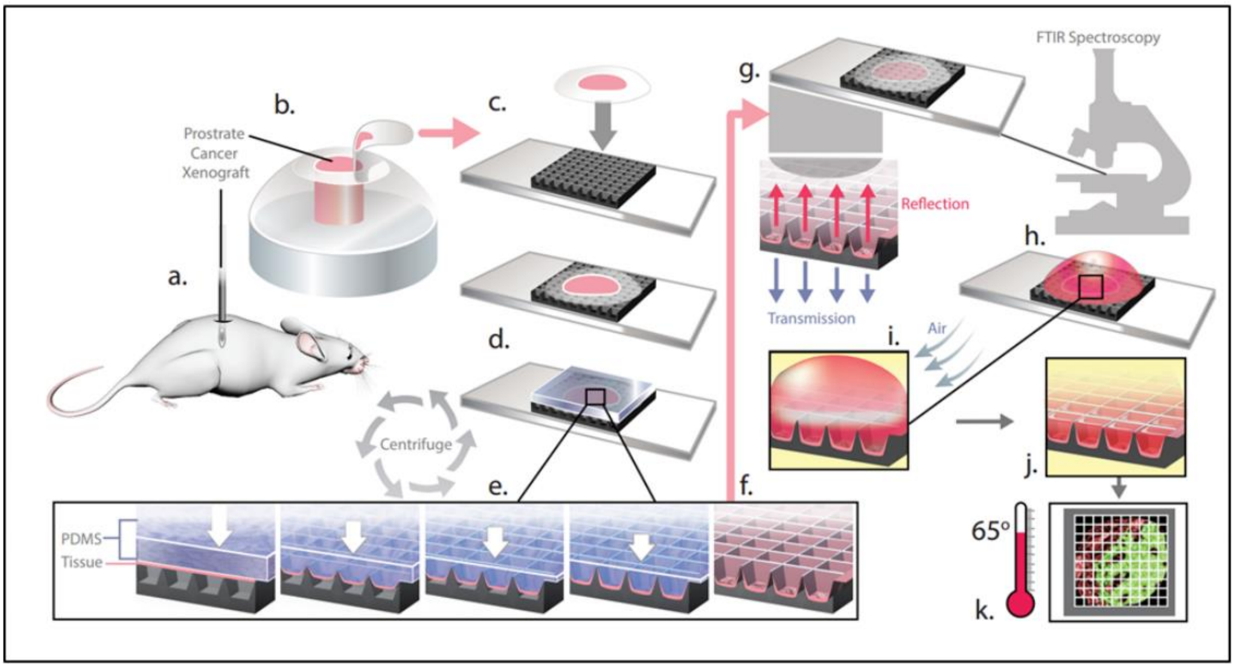

Supplementary Figure 9 | Overall process flow schematic with FTIR control on same section. **a,** LNCaP cells are injected into a mouse and prostate cancer xenograft obtained. **b,** Xenograft is resected and immediately frozen and embedded in optimal cutting temperature compound (OCT) **c,** A 7um tissue cryosection is loaded onto our microchip. **d,** A cured PDMS block is loaded on top of tissue-chip assembly. **e-f,** The PDMS shears and partitions the tissue into small pixels at sharp well edges and pushes them into wells under centripetal force in a standard centrifuge. The pixelated tissue adheres to the silanized (APTES) well surfaces and the PDMS is removed. We call this process “Tissue pixelation” (Time = 2 minutes). **g,** Post pixelation, the tissue is fixed with acetone (Time = 10 minutes). After fixation, FTIR imaging is performed on the tissue. A proteinase K digestion is performed after this to create a pathway for amplification enzymes to reach the target nucleic acids inside cells. (Time = 30 minutes). **h,** RT-LAMP reagents are pipetted on chip in bulk (5ul). **i,** Compressed air is blown on it at an angle inside mineral oil. **j,** Excess reagents are sheared away and fluid only inside wells is retained due to capillary forces. In the above steps, picoliter volume RT-LAMP reagents (~175pL/well) are loaded onto the chip through a rapid instrument-free technique we call “bulk picoliter reagent loading”. (Time = 2 minutes) **k,** Quantitative gene expression is visualized through real-time imaging of the amplification reaction in each well performed using only a hot plate at 65°C and a fluorescence microscope. (Time = 45 minutes). Images created by Janet Sinn-Hanlon, The DesignGroup@VetMed, University of Illinois at Urbana Champaign.

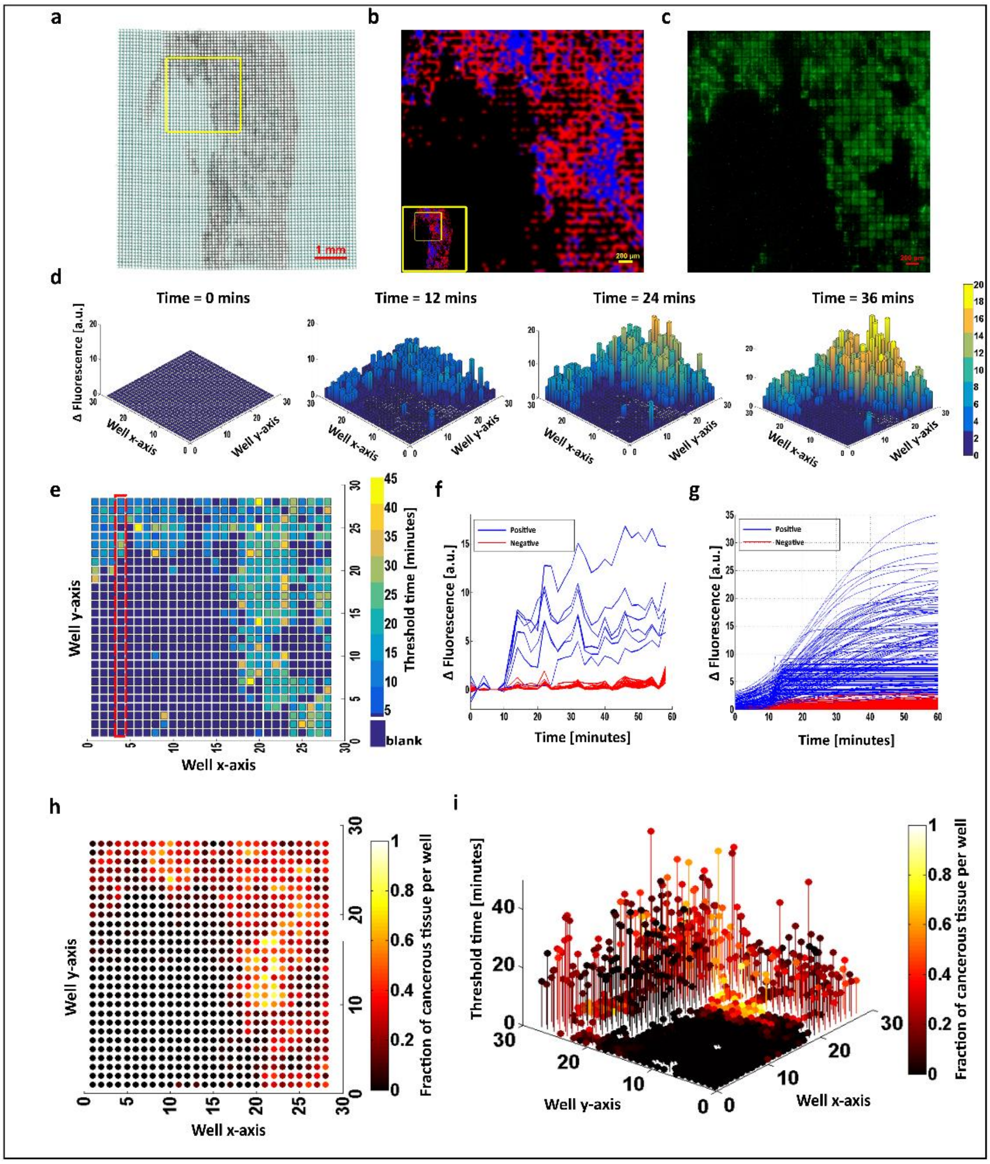

Supplementary Figure 10 | RT-LAMP with FTIR control on same section, experiment 1. a, Bright-field image of the pixelated tissue fixed on chip. The region in yellow square is the region imaged real-time during RT-LAMP **b,** FT-IR spectroscopy of our region of interest with the FT-IR image of the whole tissue in the inset. The red colour denotes the cancerous regions (epithelial) and the blue colour indicates the non-cancerous (stromal) regions. (scale bar on the bottom right is 200um) **c,** End-point fluorescence image (t=36 mins) of the region of interest after the on-chip RT-LAMP. (scale bar on the bottom right is 200um) **d,** 3D plots showing the spatial distribution of fluorescent intensity in the tissue during different times of the RT-LAMP reaction. **e,** Spatial map showing the threshold times for amplification across the imaged tissue region. **f,** Raw amplification curves from a single column of wells (marked in red in **c**) showing the clear distinction in fluorescence between positive and negative wells. **g,** Processed amplification curves from all the 784 wells imaged real-time. A clear distinction between the positive and the negative wells is observed. **h,** The fraction of cancer tissue per well as indicated by the red portion of FT-IR image in b, and **i,** a 4-D plot combining the spatially mapped threshold times with the fraction of cancer tissue per well and showing heterogeneity in TOP2A expression across the imaged tissue region. It can be observed that wells with no to negligible fraction of cancerous tissue (dark red) either don't amplify or have very high threshold times, whereas the regions with high fraction of cancerous tissue (yellow) tend to show lower threshold times.

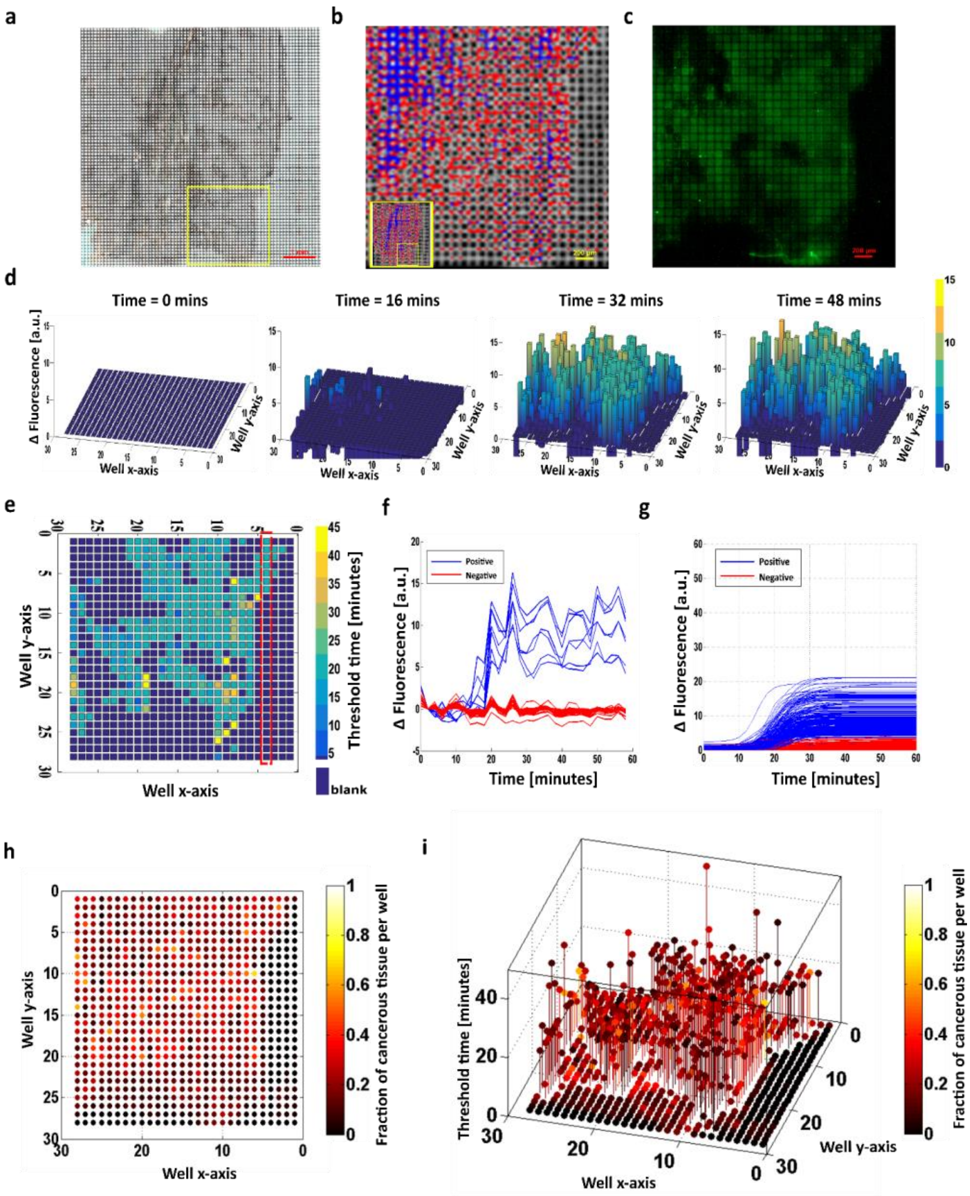

Supplementary Figure 11 | RT-LAMP with FTIR control on same section experiment 2. a, Bright-field image of the pixelated tissue fixed on chip. **b,** FT-IR spectroscopy of our region of interest with the FT-IR image of the whole tissue in the inset. The red colour denotes the cancerous regions (epithelial) and the blue colour indicates the non-cancerous (stromal) regions. **c,** End-point fluorescence image (t=48mins) of the region of interest after the on-chip RT-LAMP. **d,** 3D plots showing the spatial distribution of fluorescent intensity in the tissue during different times of the RT-LAMP reaction. **e,** Spatial map showing the threshold times for amplification across the imaged tissue region. **f,** Raw amplification curves from a single column of wells (marked in red in **c**) showing the clear distinction in fluorescence between positive and negative wells. **g,** Processed amplification curves from all the 784 wells imaged real-time. A clear distinction between the positive and the negative wells is observed. **h,** The fraction of cancer tissue per well as indicated by the red portion of FT-IR image in b, and **i,** a 4-D plot combining the spatially mapped threshold times with the fraction of cancer tissue per well and showing heterogeneity in TOP2A expression across the imaged tissue region. It can be observed that wells with no to negligible fraction of cancerous tissue (dark red) either don't amplify or have very high threshold times.

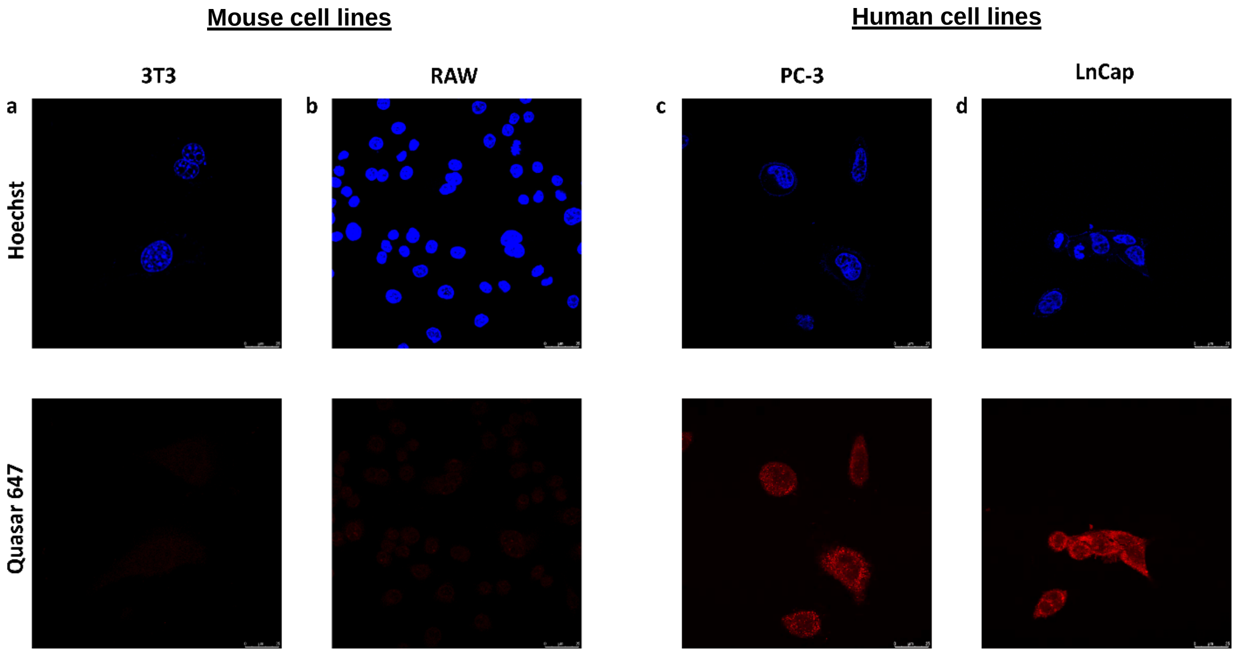

Supplementary Figure 12 | Specificity validation of TOP2A mRNA FISH in cultured cell lines. Fluorescence micrographs show nuclear stain (Hoechst; top row) and TOP2A mRNA FISH (Quasar 647; bottom row). **a-b)** TOP2A-negative mouse 3T3 fibroblasts and RAW 264.7 macrophages show no significant TOP2A mRNA FISH signal. **c-d)** TOP2A-positive human prostate cancer cell lines PC-3 and LNCaP show significant TOP2A mRNA FISH signal. The scale bar on the bottom right of each image is 25 micrometers.

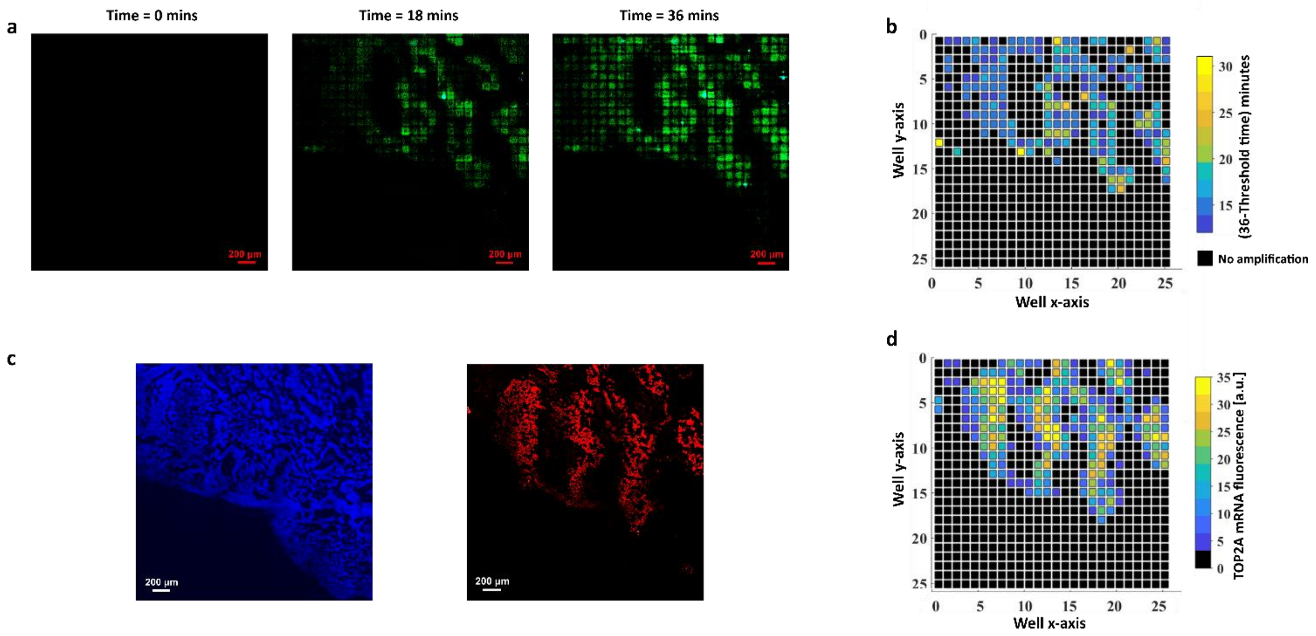

Supplementary Figure 13 | On-chip RT-LAMP with mRNA FISH on serial sections experiment 2. **a,** Baseline-subtracted fluorescence images of real-time RT-LAMP with tissue on chip at three different time points showing the increase in fluorescence over time. **b,** Spatial threshold analysis showing the spatially mapped threshold times. No amplification till 36 minutes was observed. **c,** DAPI (blue) and TOP2A mRNA FISH (red) images of the consecutive section showing spatial heterogeneity in TOP2A mRNA expression. **d,** Pixelated intensity map of mRNA FISH fluorescence. The spatial pattern of TOP2A expression is similar between the two assay types.

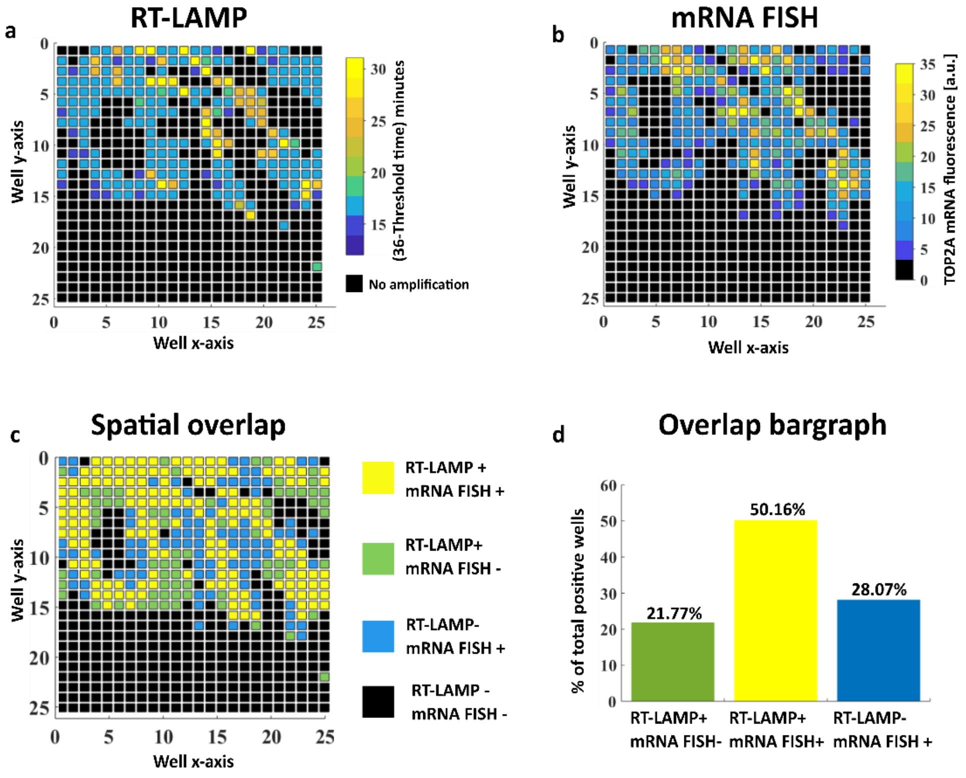

Supplementary Figure 14 | RT-LAMP - mRNA FISH overlap analysis. a, Spatial threshold map using RT-LAMP shown in Figure 6. **b,** pixelated mRNA FISH intensity map **c,** Spatial binary overlap maps of the on-chip RT-LAMP with the mRNA FISH data. The binary overlap map shows pixels with (i) both RT-LAMP and mRNA FISH signal (yellow), (ii) only RT-LAMP signal (green), (iii) only mRNA FISH signal (light blue), and (iv) neither RT-LAMP nor mRNA FISH signal (black). **d,** Bar-graph showing the different percentages of positive pixels with (i) RT-LAMP signal only (green), (ii) both mRNA FISH and RT-LAMP signal (yellow), and (iii) only mRNA FISH signal (light blue). The graph shows that the two techniques have comparable sensitivities.

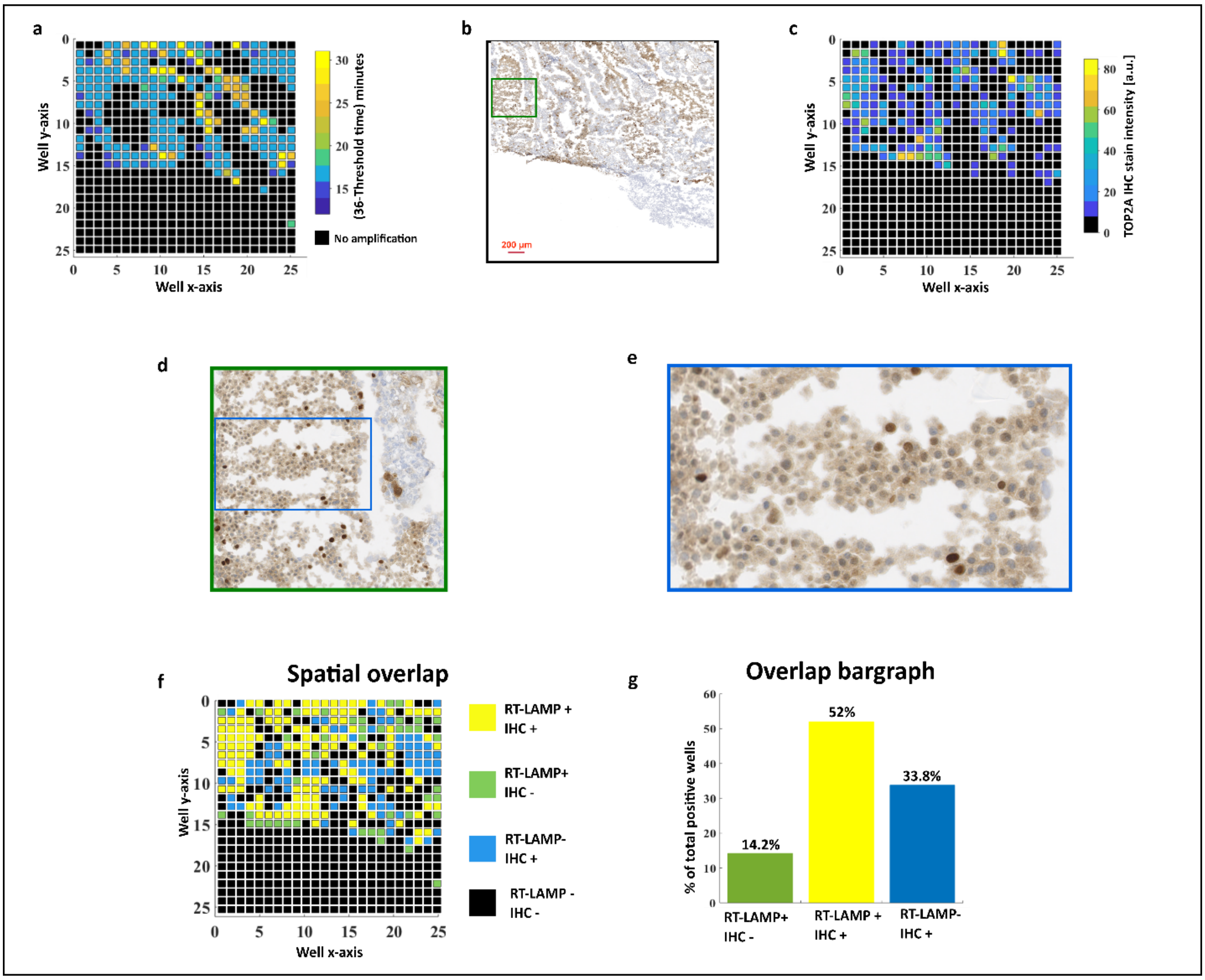

Supplementary Figure 15 | Comparison of TOP2A mRNA expression through RT-LAMP with TOP2A IHC for adjacent sections. The RT-LAMP experiment is the same as shown in Figure 6 and IHC was performed on a serial section. **a,** Spatial threshold analysis from RT-LAMP. **b,** TOP2A IHC from adjacent section in the region of interest. **c,** Pixelated TOP2A IHC expression obtained from “b”. The spatial signatures observed in the IHC images is consistent with those observed in RT-LAMP spatial threshold map. **d-e,** Zoomed in image for IHC showing staining. **f,** Spatial binary overlap maps of the on-chip RT-LAMP with the IHC data. The binary overlap map shows pixels with (i) both RT-LAMP and IHC signal (yellow), (ii) only RT-LAMP signal (green), (iii) only IHC signal (light blue), and (iv) neither RT-LAMP nor IHC signal (black). **g,** Bar-graph showing the different percentages of positive pixels with (i) RT-LAMP signal only (green), (ii) both IHC and RT-LAMP signal (yellow), and (iii) only IHC signal (light blue). The graph shows that the two techniques have comparable sensitivities.

Supplementary Table 1: Primers

The RT-PCR and RT-LAMP primers were synthesized by Integrated DNA Technologies (IDT), and are listed below.

**TOP 2A RT-LAMP primers**	**F3:** GTC GTG TCA GAC CTT GAA
	**B3:** TAG TTC CTT TTG GGG CAG
	**FIP:** TCT GGG AAA TGT GTA GCA GGA GGC TGA TGA TGT TAA GGG CA
	**BIP:** AAC CCA GTT CCT AAA AAG AAT GTG AGT GGA GGT GGA AGA CTG A
	**Loop F** GGC TTG AAG ACA GTG GTA CAC
	**Loop B:** CAG TGA AGA AGA CAG CAG CAA
**TOP2A RT-PCR primers**	**Forward:** TGG CTG CCT CTG AGT CTG AA
	**Reverse:** AGT CTT CTG CAA TCC AGT CCT CTT

Supplementary Note 1: IR imaging

FT-IR imaging was performed using a Perkin Elmer Spotlight 400 system. The spectra were collected using a mercury-cadmium-telluride (MCT) 16-element linear array detector cooled with liquid nitrogen. The system is purged with N_2 in order to reduce the interference of water vapor and CO_2 infrared signal. The background was collected on a clear area of silicon chip at 4cm^{-1} resolution using 120 scans for each sample. The images were acquired in reflection mode with 6.25 µm x 6.25 µm pixel size and 4 cm^{-1} spectral resolution with 2cm^{-1} step size using a single interferometer scan with signal to noise ratio (SNR) exceeding 600:1 in all cases. Data was collected over the mid-infrared region and truncated for storage (750cm^{-1} to 4000cm^{-1}). Since the samples were large, (smallest dimension being at least 500 µm for every section) and irregularly shaped, each image was acquired by breaking it down to 2 by 3 smaller rectangular regions and using raster scanning of these parts. Processing time for a square section of 1mm X 1mm at 6.25 µm, starting with imaging and obtaining computational stain was about 2 hours. Each region was separately focused to avoid any error due to change in focus and the composite image was stitched back together using ENVI-IDL 4.8(Environment for Visualizing Images-Interactive Data Language).

Bayesian inference-base method:

Metrics were identified by the examination of spectra, **S**, by a trained spectroscopist from regions delineated by a trained pathologist. From the universal set of metrics, $\mathbf{M} = \{m_1, m_2, ...m_n\}$, an evaluation of pairwise error and incremental increase in classification accuracy for every class, $\mathbf{C} = \{c_1, c_2,..., c_i\}$, resulted in a subset of 2 metrics. These were further reduced to a set of 18 by leaving out one metric at a time and evaluating the resulting classification accuracy on validation array data. The classification process reported here consists of evaluating the maximum *a posteriori* probability for every class c_i, $p_i(c_i|\mathbf{M})$, for every metric profile from every spectrum from the image $\mathbf{M}(x,y)$ to formulate a decision rule

$$p(c_j|\mathbf{M}) > p(c_i|\mathbf{M}), i = 1, 2, ..., n_c, i \neq j \qquad (1)$$

Classification evaluation:

As the threshold acceptance value determines an operating point for the algorithm, a systematic variation of the acceptance threshold can be used to carry out validation and statistical analyses of the classification results following methods in DC Fernandez, R Bhargava, SM Hewitt, IW Levin Nature Biotechnology 23, 469 - 474 (2005)[1].

Supplementary Table 2: mRNA FISH probes for TOP2A mRNA

	**Sequence**
**1**	TGTTACGGAGTCACTCTTTT
**2**	AGTTGAAGGTTGGTCCAGAA
**3**	ACCAAAGGGGCATATCAAGA
**4**	CCAAGTCTTCTTTCCACAAA
**5**	CTTCAACAGCCTCCAATTCT
**6**	TGTTCATCTTGTTTTTCCTT
**7**	ATGGTTATTCGTGGAATGAC
**8**	TTTTAGGCCTTCTAGTTCCA
**9**	TTGGCTTAAATGCCAATGTA
**10**	GATTCTGAATCAGACCAGGG
**11**	AATTACTTTCGTCACTGCTC
**12**	TGTTTCTCGTGGAGGGACAT
**13**	TAGGTGGACTAGCATCTGAT
**14**	CTTCAAGGTCTGACACGACA
**15**	GGCTTGAAGACAGTGGTACA
**16**	TCTGGGAAATGTGTAGCAGG
**17**	ACTGGGTTTGTAATTTCAGT
**18**	AGTGGAGGTGGAAGACTGAC
**19**	CAAAGCTGGATCCCTTTTAG
**20**	GCTTTTGAGAGACACCAGAA
**21**	ATTCTTGGTTTTGGCAGGAT
**22**	GGATTTCTTGCTTGTGACTG
**23**	ATGGAAGTCATCACTCTCCC
**24**	CCACAGCTGAGTCAAAGTCC
**25**	TTAAAACCAGTCTTGGGCTT
**26**	TTGGGCTTTACTTCACTTTG
**27**	CCATGAGATGGTCACTATTT
**28**	GCTGAAGTGATCAGATAGCT
**29**	TGCTCTATCTCATATCTACT
**30**	GAGTATCTGTACTAGAACCA
**31**	TTGGCACATAAGAGGCTGAG
**32**	TGAGCAATTTCTCATTGCTT
**33**	GGCCTCTGATGATTTGAGAA
**34**	AAATTGGTTTCTCTCTTTGG
**35**	CTTGGATCAAATGTTGTCCC
**36**	ATTGCTGAGCATGGTTATCA

Supplementary Note 2:

RT-LAMP - mRNA FISH overlap and sensitivity comparison with different grid position for mRNA FISH pixelation.

The pixelation of mRNA FISH was done by placing an artificial grid and taking the average of neighbouring pixels within one unit of the grid. Since the position of the grid was visually aligned, we shifted the grid in x (column shift) and y (row shift) in steps of 5um to obtain the best possible alignment. For each of the grid placements, mRNA FISH pixelation was performed and a binary overlap (signal positive or signal negative) with RT-LAMP was determined. Also, a ratio of the positive pixels from RT-LAMP to positive pixels from mRNA FISH was calculated.

The following parameter was used:

Positive pixel ratio = Total number of positive pixels from RT-LAMP/total number of positive pixels from mRNA FISH (indicative of relative sensitivity).

The result for this analysis is shown in **Supplementary Figure 13.**

Supplementary References

1. Fernandez, D. C., Bhargava, R., Hewitt, S. M. & Levin, I. W. Infrared spectroscopic imaging for histopathologic recognition. *Nat. Biotechnol.* **23,** 469–474 (2005).
