# Supplementary material for: Pixelated spatial gene expression analysis from tissue
Source: Nat Commun. 2018 Jan 15;9:202. doi: 10.1038/s41467-017-02623-9 (PMC5768672; doi:10.1038/s41467-017-02623-9)
Supplement: Supplementary file 2 — Peer Review File [file 41467_2017_2623_MOESM2_ESM.pdf]

Reviewers' comments:

Reviewer #1 (Remarks to the Author):

The paper by Ganguli et al presents a technique to perform on-chip pico-liter RT-LAMP on histological samples using the TOP-2A gene. The paper is well written and clear and includes clear advancements to previous papers. The authors use a 'simple' principle on pressing a single tissue section onto an array of micro-wells in which reactions are performed to maintain the spatial information of the nucleic acids.

Major comments

It is unclear if RT-LAMP will have a practical impact and some additional benchmarks will be necessary to prove the value in a diagnostic setting. The spatial component that the authors is addressing is indeed important and frequently lost in current molecular analysis of bulk samples that yields an average view of gene expression. There are three levels of targeting gene expression: whole-transcriptome, panel or single genes. A benchmark should include at least other assays at the same 'level'. In this case single gene analysis and then the benchmark should include an orthogonal method of single biomarker in tissue sections. Indeed the increased sensitivity with multiple primers is benchmarked towards a single primer set-up is valid but is still more of an incremental improvement.

• For single gene detection it would be appropriate to compare the RT-LAMP data with IHC data/in situ on the same gene on a consecutives section to confirm the heterogeneity of TOP2A gene expression. Can contaminating RBCs cause an non uniform pattern?

Another important aspect is the quality of the spatial information. From the imaging perspective you would like to study tumor cells in a context of other cells (cancer in situ, stroma, inflammatory cells etc). The suggested approach is pixelates the tissue section onto the wells and the tissue section is fragmented. The label-free imaging is 'secured' by Fourier transform infrared (FT-IR) spectroscopy and the histological output data appears not to be compatible with standard HE staining that allows for in depth histological analysis. FT-IR appears to provide a rather coarse view of tumor vs non-malignant cells in the tissue samples and it is difficult to assess the granularity in the current manuscript

• A benchmark between a HE stain and Fourier transformed images need to be included to convince a reader that information is not lost. For example how well does the classified image in Supp 8 (f) correspond to an HE stain.

• A high resolution image of a part of Figure 6 A would aid the judgement of the histology.

Minor comments

• Is the approach compatible with FFPE material?

• Are there possibilities to increase the number of targets (panel) by increasing dyes/label strategies.

• The technology is demonstrated using xenographs in mice – it would be important to demonstrate the technology on human prostate cancer samples. Is tissue pixilation possible in samples of more complex nature (connective tissue/stroma/fibrous tissue)?

Reviewer #2 (Remarks to the Author):

The manuscript of Ganguli et al. describes a novel method for analyzing spatial expression of a single gene in histological sections of prostate cancer xenografts. The authors use reverse transcription LAMP (loop mediated isothermal amplification) that allows reverse transcription of mRNA followed by real time amplification method suited for tissue analysis. This LAMP method was first described in 2000 (Ref 12). The same group as the one presenting the work here has applied this technology to the detection of HIV particles in the blood using RT-LAMP imaging the kinetics of amplification on a smartphone (Ref. 11).
Although the manuscript is mostly clearly written, some aspects are not convincingly addressed. One experiment is lacking which would demonstrate correlation between TOPO2 gene expression on the xenograft and immunostaining with TOPO2 antibody either on the same section or on an adjacent section. It would thus be important to know if the method is compatible with immunostaining. This work presents the results from the expression of one single gene. Could multiplexing be applied? Some details are missing for allowing researchers to reproduce the work.

Major points:
1-The first part of the results should be modified. Y-axis Fig.2b is not correct. It should be number and not minute. How one can compare threshold cycle numbers and threshold times in minutes? X-axis Fig.2B there is twice 10000 cells. It would be easier to present amounts of RNA instead of concentrations. Correlation coefficients should be added on all curves of Fig. 2 and Sup Fig.2. Calculations should be provided for comparing RT-LAMP and RT-PCR with thresholds and amounts of RNA. On the same line, the inhibitor effect as stated line 72 is probably the effect of inhibitors present in tissue? In that case, the result is provided by comparing Fi.2a and Sup Fig.2. Remarkably 10000 cells are equivalent to 940ng RNA per reaction and 1x tissue to 2450ng (2.6x more). The corresponding threshold times show a difference (again the reviewer does not have the precise data) of about 1 maximum which would be quite remarkable indicating very light effect of tissue inhibitors? Sup Fig.2 please give amounts of RNA.
The comparison of Fig.2a and Fig.2C gives the difference of amplification between cells and purified RNA. It is not clear how the authors could spike 1 cell in their reaction (line 72). 100 cells Fig.2a gives around 23 minutes threshold and Fig.2C 19.5. Does it mean that RT-LAMP on cells is more efficient than on RNA extracted from cells? Is it the RNA extraction? This difference should be discussed. Also RT-LAMP curves are different from RT-PCR with a peak at the start of the plateau. Fig.2b blank (black) is amplifying. 10000 cells is not very efficiently amplifying. Is there a mistake in this figure? Line 74-75 is indicating that a single cell contains cell lysate. This is obvious and the amount of cell lysate is not the same as with more cells. Again the comparison is not adequate (see above). Sentence lines 75-78 suggests that there will be 100,000 more putative tissue inhibitors in a microchip well, this should be taken into account for the sensitivity.

2-The second part of the results on pixilation of tissue indicates that there is no tissue left on the edges of the chip. This is not obvious from Fig.3e that reveals blue lines in some part of the chip. It would be important to know which region of the chip has been zoomed in Fig.3b,d,f,h from Fig.3a,c,e,g. Please indicate and move the scale bars from Fig3a,d they are not readable. Line 86 please detail APTES line 88 SEM. It would be also important to have a quantification of the nuclei along the wells in order to know if the tissue is deformed at some places. It is not clear whether PDMS is deposited as liquid or solid and if the surface are plasma treated. Fig.3g indicates more fluorescence in the region of the chip without tissue. Does it mean that the volume filed is higher and how is it possible to control the volume of liquid in the wells containing tissue? Fig.3 legend: Replace last line Extended Data Fig.1e by Sup Fig.1e. Sup Fig1a: nothing is really visible and there is no scale bar. Sup Fig.1e-f how many chips were analyzed? What will be the variation of volume? Could the authors give the correspondence between fluorescence intensity and volume? What is the range of thickness of tissue possible to use? It is hard to understand how the liquid is retained while adding the oil.
3-Real-time microchip RT-LAMP: this part demonstrates the amplification of TOP2A in regions of tissue with cancerous cells. Fig. 4a indicates fluorescence signal at time 0, could the authors comment? The curves in Fig.4e are somewhat different from the curves in Fig.2a without or with

the peak at the plateau respectively. Any comment? It would be interesting to deduce the number of cells per well from the RT-LAMP assays and comparison with the curves Fig.2.Sup Fig.4 legend indicates end-point of the RT-LAMP reaction at 48 min but line 11 is indicating 35 min, why is there a difference? Sup Fig.4 legend there is no red line in c and i description is indicated under g. Sup Fig.4 appears in the text before Sup Fig.3. Same for Sup Fig.7 that appear after Sup Fig.3. Sup Fig.3e and f are not easily readable. Is the threshold calculation method based on the QPCR method? Fig.5 it is not specified the number of experiments done (n?). In the legend bracket is missing before b.
4-FTIR: Fig.6 red line in c is not there as in Sup Fig.4.
5-Discussion: please provide numbers line 164. P.169-170 there are no data provided. Same for line 172-174 no data.

Minor points:
The numbering of the Figures should be verified, figures should appear with increasing numbers in the text.
References 11 and 27 are the same.
Reference 28 is not available
Method section: cells and xenografts: please rephrase this paragraph and add the steps one by one. Please indicate what OCT means?

Reviewer 1:

1. ***It is unclear if RT-LAMP will have a practical impact and some additional benchmarks will be necessary to prove the value in a diagnostic setting. The spatial component that the authors is addressing is indeed important and frequently lost in current molecular analysis of bulk samples that yields an average view of gene expression. There are three levels of targeting gene expression: whole-transcriptome, panel or single genes. A benchmark should include at least other assays at the same 'level'. In this case, single gene analysis and then the benchmark should include an orthogonal method of single biomarker in tissue sections. Indeed the increased sensitivity with multiple primers is benchmarked towards a single primer set-up is valid but is still more of an incremental improvement. For single gene detection, it would be appropriate to compare the RT-LAMP data with IHC data/in situ on the same gene on a consecutives section to confirm the heterogeneity of TOP2A gene expression. Can contaminating RBCs cause a non-uniform pattern?***

   Response: We appreciate the reviewer's comments and are pleased to have this opportunity to address them. With regard to the first comment, the reviewer is correct in that LAMP is more specific due to the use of the 6 primers and hence works better in tissue or in complex samples, as compared to PCR. Hence, LAMP is an essential component of our experiments because of the robustness of this technique. In Figure 2, we have demonstrated that RT-LAMP is at least an order of magnitude more robust than RT-PCR. In these experiments in which RNA amplification must be carried out in the presence of cellular debris and without a separate step of RNA isolation, the increased robustness afforded by the RT-LAMP is crucial to the success of the newly proposed approach.

   Regarding the validation with another technique – this is an excellent point and we have now added significant additional materials to address this important and useful comment. To confirm the heterogeneity of TOP2A gene expression and validate our RT-LAMP data, we have performed mRNA fluorescence in-situ hybridization (FISH) on consecutive 7um thin sections from the same tissue sample. The results of these experiments are shown now in Figure 6 of the main manuscript. The signature for TOP2A mRNA expression in the tissue is preserved across the serial sections and can be observed in both mRNA FISH and our microchip amplification pixelated images. Hence, this new control now validates our technique.

   Regarding the reviewer's last question, we do not expect RBCs to be interfering with the TOP2A RT-LAMP reaction. We have shown previously that the RBCs in the blood do not interfere with the RT-LAMP reaction[1]. And now with the new additional data and the control experiments using FISH validates and confirms the results of the amplification, further proving that the cellular debris and contaminants including RBCs do not cause an issue.

2. ***Another important aspect is the quality of the spatial information. From the imaging perspective, you would like to study tumor cells in a context of other cells (cancer in situ, stroma, inflammatory cells, etc.). The suggested approach pixelates the tissue section onto the wells and the tissue section is fragmented. The label-free imaging is 'secured' by Fourier transform infrared (FT-IR) spectroscopy and the histological output data appears not to be compatible with standard HE staining that allows for in depth histological analysis. FT-IR appears to provide a rather coarse view of tumor vs non-malignant cells in the tissue samples and it is difficult to assess the granularity in the current manuscript.***

Response: We agree with the reviewer's comments. The information content of IR imaging is a topic of ongoing research in other researcher groups. While the information content in IR spectra allows the possibility to reproduce H&E stains[1], for our work we only performed FTIR as a control on the same tissue section to differentiate between cancer and non-cancer cells. So it was meant as a crude initial control. The FTIR approach currently also takes a long time to complete the measurement and the analysis, and while researchers are working to reduce that time, in the implementation plans of the pixelated amplification technology demonstrated in this paper, FTIR will NOT be included in the actual implementation flow in the clinics.

We have modified the manuscript to indicate this clearly and moved the discussion on the FTIR as supplementary materials. Our novelty is the on-chip RT-LAMP assay introduced here which allows amplification of nucleic acid molecules from an entire tissue section without analyte purification and while preserving spatial information. This has not been demonstrated previously.

3. ***A benchmark between a HE stain and Fourier transformed images need to be included to convince a reader that information is not lost. For example how well does the classified image in Supp 8 (f) correspond to an HE stain.***

   Response: By combining optical and IR information, FTIR achieves a high accuracy in morphological classification of cells in tissue sections. Our previous work has already benchmarked FTIR in cross validation studies that included different staining conditions (including H&E) with area under ROC curves in excess of 0.95[2]. In the current project, FTIR was only used as a crude control on the same section of tissue and will not be part of the final implementation of the technique. Besides, as noted above, while possible compatibility of the FTIR technique with our approach is attractive, FTIR does not allow mRNA expression or DNA quantitation and hence is not part of our implementation plan. We appreciate the reviewer's point and realize that we did not emphasize this earlier and we have modified the document to reflect this clearly. The FTIR experimental results have been moved to the supplementary section and the manuscript text is modified to explicitly mention FTIR only as a partial control of epithelial versus stroma, and not a control for the amplification itself. In this this paper, the real novelty is the spatially preserved nucleic acid amplification methodology which can be accomplished in less than 2 hrs., which we have now further validated using the mRNA fluorescence in-situ hybridization

Minor comments:

4. ***Is the approach compatible with FFPE material?***

   Response: We expect that our procedure will be compatible and we are in the process of optimizing the process with FFPE tissue. Once the FFPE tissue is deparaffinized on the microchip, we expect our protocol to work very similarly to the tissue cryosection protocol. We appreciate the reviewer's interest in this question and realize the added potentials in clinical applications once our procedures are made consistent with FFPE tissue. This will be included in our next paper.

5. ***Are there possibilities to increase the number of targets (panel) by increasing dyes/label strategies?***

   Response: Yes, there are possibilities of increasing the number of targets analyzed per well by using a multiplexed lamp approach. The primers could be spotted in advance or introduced later

when the reagents are added. Several demonstrations of multiplexed lamp have been shown in the literature[3–5] and we are working to integrate these with our on-chip process from tissue in our subsequent studies.

6. ***The technology is demonstrated using xenografts in mice – it would be important to demonstrate the technology on human prostate cancer samples. Is tissue pixelation possible in samples of more complex nature (connective tissue/stroma/fibrous tissue)?***

Response: During the process of testing and optimization of our tissue pixelation approach, we have tested highly fibrous tissues, such as rat heart and also soft tissues such as rat brain. These tissues were all successfully pixelated. We have added supplementary Fig. 3 for SEM images of Rat heart tissue pixelation in response to this comment. Also, in the experiment for Figure 5 of the paper (Cancer vs non-cancer control), we pixelated mouse skeletal muscle tissue in addition to the prostate cancer xenograft tissue on the same chip.

Reviewer 2:

1. **Although the manuscript is mostly clearly written, some aspects are not convincingly addressed. One experiment is lacking which would demonstrate correlation between TOPO2 gene expression on the xenograft and immunostaining with TOPO2 antibody either on the same section or on an adjacent section. It would thus be important to know if the method is compatible with immunostaining. This work presents the results from the expression of one single gene. Could multiplexing be applied? Some details are missing for allowing researchers to reproduce the work.**

   Response: To confirm the heterogeneity of TOP2A gene expression and validate our RT-LAMP data, we have now performed mRNA fluorescence in-situ hybridization (FISH) on consecutive sections. We decided that FISH would be more compatible as a control with our experiment than IHC since the correlation between mRNA and protein is not always perfect. As FISH is also labeling the mRNA, this is a more appropriate comparison. The results of these experiments are shown in Figure 6 of the revised manuscript. The signature for TOP2A mRNA expression in the tissue is preserved across the serial sections and can be observed in both mRNA FISH and microchip amplification pixelated images. The correlation between the two techniques are highly significant with spearman correlation of -0.56 (p value = 8.7881e-55) for Figure 6 panel 1 and -0.57 (p value= 3.9886e-54) for Figure 6 panel 2**.** The addition of FISH significantly improves the manuscript and we really appreciate this reviewer's comment in steering us in that direction.

   Multiplexed LAMP can also be employed with our technique to increase the number of targets analyzed per well. Several demonstrations of multiplexed lamp have been shown in the literature[3–5] and we are working to integrate these with our on-chip process from tissue in our subsequent studies in future paper. We believe that this paper has already enough depth and breadth for the first paper on this technology.

   The details about the off-chip reactions protocol are mentioned in the methods section under "offchip reactions". For the on-chip reaction protocol, the methods sections "Chip Fabrication and Chip Silanization", "Tissue Pixelation, Fixation, and Proteinase K digestion", "Bulk Loading and On-chip RT-LAMP reactions" and "Amplification Data Analysis" describe a detailed process flow for different steps. In addition to this, the times required for each step have been added in the main manuscript as well as in the Figure 1 caption. Details about the mRNA in-situ hybridization technique on consecutive sections have also been added in the Online methods section.

2. ***The first part of the results should be modified. Y-axis Fig.2b is not correct. It should be number and not minute. How one can compare threshold cycle numbers and threshold times in minutes?***

   Response: We thank the reviewer for the careful review. The correction is made in Figure 2b from "Threshold cycle [minutes]" to "Threshold cycle [number]". Also, a time axis in minutes has been added on the top of the Figure 2b to allow for visual comparison of reaction times between RT-LAMP and RT-PCR. For RT-PCR, time of amplification in minutes was found by multiplying the threshold cycle number with the time taken for each thermal cycle, which is a constant for a system or protocol. Each cycle in our RT-PCR recipe had a 15s denaturation step at 95C and a 30s combined annealing and elongation step at 60C. Additionally, there was a ~35s ramp time in each cycle resulting in 80s (~53 minutes/40 cycles) for the completion of one cycle. The thermocycler protocol is shown below for the convenience of the reviewer.

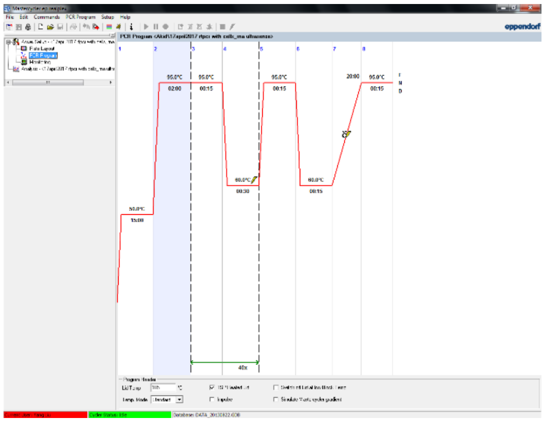

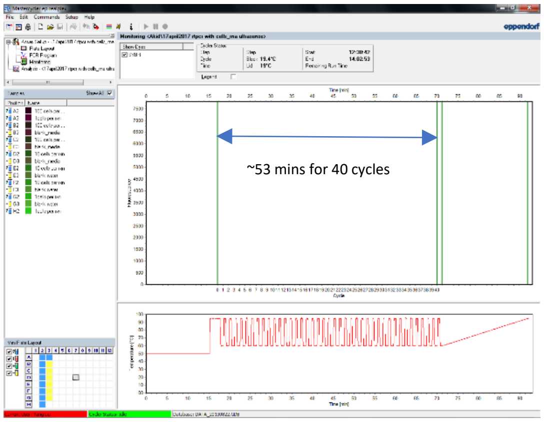

3. ***X-axis Fig.2B there is twice 10000 cells. It would be easier to present amounts of RNA instead of concentrations. Correlation coefficients should be added on all curves of Fig. 2 and Sup Fig.2. Calculations should be provided for comparing RT-LAMP and RT-PCR with thresholds and amounts of RNA.***

   Response: The correction to X-axis Fig.2B is made in the document. The total amount of the purified RNA for all the amplification reactions have been included in the figure captions as suggested for clarity. The calculation of threshold times for both RT-PCR and RT-LAMP has been described in the methods section under "Amplification Data Analysis". When comparing RT-LAMP and RT-PCR, we are only concerned about the limits of detection of the two assays. Since the total amount of RNA/cells have been kept constant across the two assays, we are able to compare the limits of detection of the two assays directly. We have added the line "The amounts of RNA per reaction for each dilution was the same as in RT-LAMP (Figure 2a) to allow direct comparison. " in the Figure caption description of Figure 2b. Also, the line "The total amounts of RNA per reaction for each dilution was identical in the RT-LAMP and RT-PCR reactions for direct comparison of the two techniques." Has been added in the main manuscript in "TOP2A mRNA RT-LAMP in a thermocycler" results section.

4. ***In that case, the result is provided by comparing Fig.2a and Sup Fig.2. Remarkably 10000 cells are equivalent to 940ng RNA per reaction and 1x tissue to 2450ng (2.6x more). The corresponding threshold times show a difference (again the reviewer does not have the precise data) of about 1 maximum which would be quite remarkable indicating very light effect of tissue inhibitors? Sup Fig.2 please give amounts of RNA.***

   Response: Off-chip amplification reactions from cultured LNCaP cells and from LNCaP xenograft tissue cryosections are 2 independent experiments where 1x of tissue refers to RNA purified from a single 7um thick tissue cryosection (This information has been added in the updated Sup. Fig 2 caption). The amount of RNA purified from the cryosection will depend on its 2D spatial dimensions. The threshold time (minutes) for 10000 cells (940ng RNA) was 13.27 ± 0.17 and for 1x tissue (2450 ng RNA) was 11.76 ± 0.3, resulting in a difference of 1.5 minutes between the two. Additional divisions in the threshold time axis have been shown in the updated figures for better time estimations. Indeed, the reviewer is correct in pointing out that there is very light effect of

tissue inhibitors observed which we believe is the result of the RNA purification step prior to the amplification reaction

5. ***The comparison of Fig.2a and Fig.2C gives the difference of amplification between cells and purified RNA. It is not clear how the authors could spike 1 cell in their reaction (line 72). 100 cells Fig.2a gives around 23 minutes threshold and Fig.2C 19.5. Does it mean that RT-LAMP on cells is more efficient than on RNA extracted from cells? Is it the RNA extraction? This difference should be discussed.***

   Response: Cultured LNCaP cells were counted using a hemocytometer and serial dilutions were prepared down to 1 cell per final reaction. This information has been updated in the document on page 2 as "using hemocyotemeter counting and serial dilutions". Yes, the reviewer is correct in noticing that RT-LAMP on cells works better than on RNA extracted from cells for up to 100 cells in reaction. We believe there is some loss of RNA in the purification process associated with the commercial RNA purification kit's efficiency. Information on purification efficiency of RNeasy purification kit was not available from the manufacturer. We have added this comment on page 2- "As seen from Figure 2a and Figure 2c, the amplification reaction works better for 100 cells spiked directly into the reaction as compared to purified RNA from 100 cells. We believe that this is due to the loss of some RNA during the RNA purification process."

6. ***Also RT-LAMP curves are different from RT-PCR with a peak at the start of the plateau.***

   Response: The reviewer is correct in pointing out the differences between the amplification curves of the two assays. Although, the reason behind this is not very clear, this phenomenon has been observed previously in LAMP reactions.[6–8]. This characteristic peak before the plateau has also been observed by the manufacturers (New England Biolabs) of Bst Polymerase used in LAMP when characterizing the enzymes activity as can be seen from the link below.
   https://www.neb.com/products/m0538-bst-20-warmstart-dna-polymerase

7. ***Fig.2b blank (black) is amplifying. 10000 cells is not very efficiently amplifying. Is there a mistake in this figure?***

   Response: The experiment for the Figure 2b has been repeated and the Figure 2b has been replaced in the updated document. In the updated figure, the 10,000 cells reaction amplifies efficiently, and blanks remain low in intensity. The Limit of Detection was again obtained as RNA equivalent to 100 cells. In addition to the RT-PCR experiment from purified RNA, we also performed RT-PCR with spiked cells (1-100) in reaction tubes. There was no amplification observed (Data shown below). For the corresponding RT-LAMP experiment with spiked cells (Figure 2c) we saw good amplification results. This confirms the robustness of Bst polymerase to inhibitors such as cell debris as opposed to Taq DNA polymerase used in PCR.

RT-PCR with cells spiked in reaction

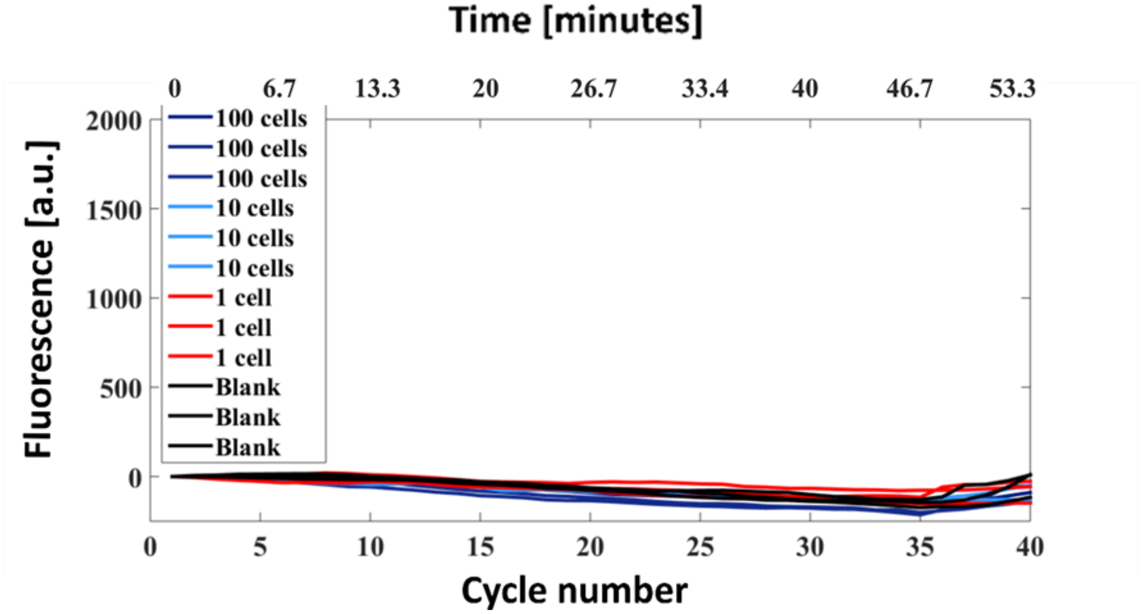

8. ***Line 74-75 is indicating that a single cell contains cell lysate. This is obvious and the amount of cell lysate is not the same as with more cells. Again the comparison is not adequate (see above). Sentence lines 75-78 suggests that there will be 100,000 more putative tissue inhibitors in a microchip well, this should be taken into account for the sensitivity.***

    Response: The reviewer is correct in pointing out that the increase in analyte concentration would be accompanied by a corresponding increase in the tissue contaminant concentration for our on-chip reactions. However, our technique was designed to avoid the tissue contaminants from being an effective part of the reaction mix in wells. As opposed to lysing the whole tissue, in which scenario, the tissue debris would have been completely mixed with the overlying solution, we chose a gentler process of proteinase K digestion to expose the target analyte inside the tissue. As can be seen from Figures 4, 5, and 6, the tissue structure is preserved in our process and from Figure 3, it can be seen that the tissue rests at the bottom of the wells. We believe that the reaction initiates due to the diffusion of enzymes into the tissue through the pathway created by proteinase K digestion, followed by diffusion of amplicons to the solution and reaction in solution in wells. During this entire process, the tissue debris/chassis which remains at the bottom of the wells does not interfere with the reaction in solution and behaves like a precipitate. Hence, we believe we can achieve much higher sensitivity for on-chip reactions. To reflect this idea, the following comment has been added in the manuscript: “As will be shown in later sections, for our on-chip reactions from tissue, the tissue debris remains attached to the bottom of the wells and does not become a part of the reaction in the solution above. This causes the effect of tissue contaminants on the reaction in wells to be negligible.” Another comment while discussing proteinase k digestion on page 3 has been added – “As opposed to lysing the whole tissue, in which scenario the tissue debris would have been completely mixed with the overlying solution in wells, using proteinase K digestion to expose the target analyte inside the tissue prevents the tissue contaminants from becoming a significant part of the reaction.”

9. ***The second part of the results on pixelation of tissue indicates that there is no tissue left on the edges of the chip. This is not obvious from Fig.3e that reveals blue lines in some part of the chip. It would be important to know which region of the chip has been zoomed in Fig.3b,d,f,h from Fig.3a,c,e,g. Please indicate and move the scale bars from Fig3a,d they are not readable.***

   Response: To validate that there is no tissue left on the well edges after tissue pixelation, the SEM image and the DAPI staining of the same pixelated tissue sample has been shown Fig 3. In Fig. 3a-d, the SEM images clearly show that there is no tissue present at the well edges. In Fig. 3e, which is DAPI stained fluorescent image of the pixelated tissue using 4X objective, the edges are clearly visible as dark lines (The grid made by horizontal and vertical black lines in the fluorescent image are due to well edges which are non-fluorescent since they don't have any tissue). Moreover, the results of this experiment are further validated in the on-chip amplification reactions downstream where tissue margins are strictly preserved throughout the amplification reaction and no cross-contamination between adjacent wells is seen at the tissue borders. The changes to scale bars and chip regions being imaged have been made in the revised manuscript.

10. ***Line 86 please detail APTES line 88 SEM.***

    Response: We thank the reviewer for the comment**.** The full form of APTES and SEM have been added in the main text at their first reference in the revised manuscript..

11. ***It would be also important to have a quantification of the nuclei along the wells in order to know if the tissue is deformed at some places. It is not clear whether PDMS is deposited as liquid or solid and if the surface are plasma treated.***

    Response: Tissue deformation inside the wells is visible in SEM images **(Fig 3 a-d)** and as can be seen from these images, some part of the tissue sticks to slanted well walls. A cured PDMS block is used to push the tissue into the underlying wells using a standard centrifuge and the sharp well edges cut the tissue into small pixels in the process. The word "PDMS" has been replaced with "Cured PDMS" in the document for clarity. The explanation "This was done by applying a centrifugal force on the tissue disc via a flexible cured polydimethylsiloxane (PDMS) block, using centrifugation in a standard centrifuge (1min@ 1500g force). When the flexible PDMS pushes its way into the wells under centrifugal forces, it shears and partitions the tissue via the sharp well edges. The tissue sticks to the well surface which was pre-silanized ((3-Aminopropyl)triethoxysilane or APTES), while the PDMS layer restores back to its original shape in the absence of force, as shown in the **Figures 1d-f**." is provided in the main text under " Tissue pixelation and bulk picoliter reagent loading".

12. ***Fig.3g indicates more fluorescence in the region of the chip without tissue. Does it mean that the volume filled is higher and how is it possible to control the volume of liquid in the wells containing tissue?***

    Response: After the compressive force applied during the tissue pixelation step, the effective change in volume of a well with tissue compared to an empty well should be negligible, as can be seen from SEM images after tissue pixelation (Fig 3a-d). As Rhodamine dye has been previously shown to stain tissue samples[9,10], we suspect that the reduced fluorescence in regions of the chip with tissue could be due to tissue-dye interactions. The purpose of our experiment was to

visualize the reagent filling on a macro scale and specifically to make sure that adjacent wells are not fluidically connected. The well edges in Fig. 3g-h can be seen as dark lines indicating they are above the level of fluorescent dye. This data, in combination with the SEM images in Fig. 3a-d, confirmed well isolation during the reaction. This was further validated downstream in the on-chip amplification reactions where tissue margins were strictly preserved and no cross-contamination was seen during amplification at the tissue borders. Since the wells are filled with capillary action, it is not possible to control individual well volume during the filling.

13. ***Fig.3 legend: Replace last line Extended Data Fig.1e by Sup Fig.1e. Sup Fig1a: nothing is really visible and there is no scale bar.***

    Response: The above-mentioned changes have been made in the revised manuscript. We thank the reviewer for pointing this out.

14. ***Sup Fig.1e-f how many chips were analyzed? What will be the variation of volume? Could the authors give the correspondence between fluorescence intensity and volume? What is the range of thickness of tissue possible to use? It is hard to understand how the liquid is retained while adding the oil.***

    Response: For Sup Fig.1e-f, 3 chips were analyzed and comparable results were observed. The data shown is from one of the chips. The exact correspondence between fluorescence intensity and volume could not be possible as there is an inherent difference in fluorescence intensity between wells with and without tissue, as discussed above in response 12. For our study, we have only used 7um thick sections. Although, we believe higher tissue thicknesses can be analyzed by using deeper wells and with minor adjustments to the protocol.

    The oil is added on top of the reagent-filled wells. The oil has a lower density than water-based reaction mix and hence it stays on top of the reagent-filled wells and prevents evaporation. The comment- “Mineral oil has a lower density than water based reaction-mix and hence it stays on top of the reagent-filled wells.” has been added in page 3 of the updated document for clarity.

15. ***Real-time microchip RT-LAMP: this part demonstrates the amplification of TOP2A in regions of tissue with cancerous cells. Fig. 4a indicates fluorescence signal at time 0, could the authors comment?***

    Response: The signal is from non-specific staining of the tissue by evergreen dye. This is a background signal and does not affect our experimental measurements as we are concerned with the change in fluorescence during the amplification reaction. As shown in Figure 4b, the change in fluorescence (Delta F) is plotted as a spatial map over time.

16. ***The curves in Fig.4e are somewhat different from the curves in Fig.2a without or with the peak at the plateau respectively. Any comment?***

Response: The curves in Figure 4e are generated after sigmoidal fitting (described in methods) to the raw data from on-chip amplification (Fig 4d) and the fitting analysis removes any possible peaks from the data and returns a smooth sigmoidal curve. The curves in Fig. 2a are raw amplification curves (without any fitting) as obtained from commercial thermocycler. In the past we have similarly observed that the off-chip (thermocycler based) amplification curves have a peak before the plateau while on-chip curves do not [6].

17. ***It would be interesting to deduce the number of cells per well from the RT-LAMP assays and comparison with the curves Fig.2***

    Response: Cells within tissue are not of uniform size, i.e. epithelial cells are much larger than lymphocytes. Therefore, there is likely to be variability in the number of cells per well depending on whether the well contains tumor, stroma, tumor infiltrating lymphocytes, etc. As a result, we chose to use FTIR data for on-chip quantification of the fraction of cancer tissue per well. As shown in Supplementary Fig 5h-i, we plotted the fraction of cancerous tissue per well (color-bar) against spatially mapped threshold times to create a 4-dimensional plot showing the spatial heterogeneity in TOP2A mRNA across the tissue.

18. ***Sup Fig.4 legend indicates end-point of the RT-LAMP reaction at 48 min but line 11 is indicating 35 min, why is there a difference?***

    Response: The reaction completion time is when all the positive reactions (wells) amplified and no further amplifications or change in fluorescence were observed after that time. We chose to monitor the reactions for a longer time (continued to take images), but all the reactions were found to be complete within the mentioned 35 minutes time span from the reaction start.

19. ***Sup Fig.4 legend there is no red line in c and i description is indicated under g. Sup Fig.4 appears in the text before Sup Fig.3. Same for Sup Fig.7 that appear after Sup Fig.3. Sup Fig.3e and f are not easily readable.***

    Response: The above-mentioned changes have been made to the revised manuscript.

20. ***Is the threshold calculation method based on the QPCR method?***

    Response: Threshold time (Ct) is a LAMP measurement analogous to quantification cycle (Cq) of qPCR. The amplification and standard curves were plotted using a MATLAB script. The threshold time for each curve was taken as the time required for each curve to reach 20% of the maximum intensity. This has been described in further detail in the methods section under the heading "Amplification Data Analysis".

21. ***Fig.5 it is not specified the number of experiments done (n?). In the legend bracket is missing before b.***

    Response: The number of experiments done with Cancer vs. non-cancer tissue loaded on the same chip were 3. Moreover, the results of these experiments were further validated by all the downstream FTIR experiments (Sup Fig 7 and Sup Fig 8) which outlined Cancer vs non-cancer regions in the same tissue section followed by amplification seen only in cancerous regions. These FTIR based experiments helped avoid the possible contamination risks associated with loading

cancer and non-cancer tissue on the same chip (Area<1cm2 for microchip) as done for the Fig. 5 experiments. The bracket before b has been added in the legend. We thank the reviewer for the detailed comments.

22. ***5-Discussion: please provide numbers line 164.***

    Response: Numbers "1-2" minutes have been added in the updated document.

23. ***169-170 there are no data provided. Same for line 172-174 no data.***

    Response: 169-170: "The same can be done for lower well sizes that go down to a single cell per well, in which case we can make the wells deeper to get similar reaction volumes as for the 100um wells to keep the same reaction kinetics."

    -This statement argues the possibility of going down to single cell spatial resolution using the same system by keeping the reaction volumes same. Since the heart of our platform is a biochemical amplification reaction, keeping similar reaction volumes will allow similar reaction kinetics in a chip with deeper wells but with better spatial resolution. Fluorescence change vs reaction volume in an amplification reaction has been studied previously [11]. We have modified the statement to "We **expect** the same should be possible for lower well sizes that go down to a single cell per well, in which case we can make the wells deeper to get similar reaction volumes as for the 100um wells to keep the same reaction kinetics."

    172-174: Possibility of deployment in resource starved settings is argued based on our previously published work where we have shown amplification in point-of-care systems using a smartphone and a portable setup. The reference for this work has been added. [6].

24. ***The numbering of the Figures should be verified, figures should appear with increasing numbers in the text. References 11 and 27 are the same. Reference 28 is not available. Method section: cells and xenografts: please rephrase this paragraph and add the steps one by one. Please indicate what OCT means?***

    Response: We thank the reviewer for the comments. The figure order has been corrected to appear in increasing numbers. References have been corrected. The cells and xenografts section in methods has been rephrased to have steps in a chronological order. The complete name of OCT – " Optimal cutting temperature compound" has been added in the document.

References:

1. Mayerich, D., Walsh, M., Kadjacsy-Balla, A. & Ray, P. Stain-less staining for computed histopathology. *Technology* (2015).

2. Kwak, J. T., Hewitt, S. M., Sinha, S. & Bhargava, R. Multimodal microscopy for automated histologic analysis of prostate cancer. *BMC Cancer* **11,** 62 (2011).

3. Kouguchi, Y., Fujiwara, T., Teramoto, M. & Kuramoto, M. Homogenous, real-time duplex loop-mediated isothermal amplification using a single fluorophore-labeled primer and an intercalator dye: Its application to the simultaneous detection of Shiga toxin genes 1 and 2 in Shiga toxigenic Escherichia coli isolates. *Mol. Cell. Probes* **24,** 190–195 (2010).

4. Aonuma, H. *et al.* A single fluorescence-based LAMP reaction for identifying multiple parasites in mosquitoes. *Exp. Parasitol.* **125,** 179–183 (2010).

5. Tanner, N., Zhang, Y. & Jr, T. E. Simultaneous multiple target detection in real-time loop-mediated isothermal amplification. *Biotechniques* (2012).

6. Damhorst, G. L. *et al.* Smartphone-imaged HIV-1 reverse transcription loop-mediated isothermal amplification (RT-LAMP) on a chip from whole blood. *Engineering* **1,** 324–335 (2015).

7. Marciniak, J., Kummel, A., Esener, S., Heller, M. & Messmer, B. Coupled rolling circle amplification loop-mediated amplification for rapid detection of short DNA sequences. *Biotechniques* **45,** 275–80 (2008).

8. Evaluation of a Portable Amplification Platform for Loop-Mediated Isothermal Amplification (Lamp) of Foot-and-Mouth Disease Virus (Fmdv) and African Swine Fever (Asfv) | OptiGene. Available at: http://www.optigene.co.uk/applications/evaluation-of-a-portable-amplification-platform-for-loop-mediated-isothermal-amplification-lamp-of-foot-and-mouth-disease-virus-fmdv-and-african-swine-fever-asfv/. (Accessed: 16th April 2017)

9. Greenspan, P., Mayer, E. P. & Fowler, S. D. Nile Red" A Selective Fluorescent Stain for Intracellular Lipid Droplets.

10. Liisberg, M. F. Rhodamine B as an extremely specific stain for cornification. *Cells Tissues Organs* **69,** 52–57 (1968).

11. Hidenori Nagai, *, Yuji Murakami, Yasutaka Morita, Kenji Yokoyama, and & Tamiya, E. Development of A Microchamber Array for Picoliter PCR. (2000). doi:10.1021/AC000648U

Reviewers' comments:

Reviewer #1 (Remarks to the Author):

I feel that the points raised in my previous round of review have been satisfactorily addressed.

Reviewer #2 (Remarks to the Author):

The revised manuscript of Ganguli et al. now addresses the important point of demonstrating that RT-LAMP correlates with another similar technique. The authors did mRNA FISH on adjacent sections and showed "substantial" correlation. Although Fig.6 looks convincing, the authors need to compare Fig. 6.1.d to 6.2.d. They should provide the comparison of intensity of pixels with both techniques. It appears that mRNA FISH is more sensitive than RT-LAMP. This new result should be discussed.
As said in the rebuttal letter, mRNA FISH is more comparable to RT-LAMP than IHC would be. However, it would be nonetheless important to correlate TOP2A gene expression to IHC used in clinic.
For this new experiment, it is not clear from the result section or the M&M section which of the list of probes in Sup Note 3 were used.

In Fig. 2 and Sup. Fig. 2 the reviewer does not see the changes made in the Threshold time axes.

The new experiment shown in Fig2b is quite different from the previous one in the original version of the manuscript. This really questions the reproducibility of this experiment.

Comment 12 should be added in the text since it will appear striking from readers of Fig. 3g.

Reviewer #2 (Remarks to the Author):

Reviewer's Comment: ***The revised manuscript of Ganguli et al. now addresses the important point of demonstrating that RT-LAMP correlates with another similar technique. The authors did mRNA FISH on adjacent sections and showed "substantial" correlation. Although Fig.6 looks convincing, the authors need to compare Fig. 6.1.d to 6.2.d. They should provide the comparison of intensity of pixels with both techniques. It appears that mRNA FISH is more sensitive than RT-LAMP. This new result should be discussed.***

Our Response: We would like to point out that in previous submission Figure 6.1.d and 6.2.d were both processed mFISH data from different sections of tumor xenograft and comparing the two would be comparing signal intensities between two mFISH experiments, not between an mFISH and an RT-LAMP experiment. To show reproducibility in the previous submission, we performed two parallel sets of RT-LAMP and mFISH experiments in panels 1 and 2. To minimize any potential misinterpretation by the readers, we have moved one panel to the supplementary material. Furthermore, we have performed additional analysis and demonstrated that based on detection threshold (not intensity), the sensitivity of RT-LAMP at an arbitrary reaction time of 36 minutes and mFISH are compatible with each other (please see Figure 1 below, which is also included as Supplementary Figure 13 in the revised manuscript) and within the range of variations between two mFISH experiments on different tissue sections (Figure 2 below for reviewer's consideration). We believe that the sensitivity of RT-LAMP can be made higher by increasing the reaction time. Furthermore, the true mFISH sensitivity is most likely less that the presented data because we did not subtract auto-fluorescence, which is known to be an issue in FISH assays. It is important to note that auto-fluorescence can vary between different tissue sections and hence it is difficult to quantitatively account for it in the test experiments for mRNA FISH.

We believe that comparing assay sensitivities based on the interpreted signal intensities (shown in previous submission Figure 6 b&d, both panels) is not appropriate as these two intensities have somewhat different meanings. While mFISH signal intensities are related to the number of target mRNA that hybridize to the probe, the RT-LAMP signal intensities are (inversely) related to the time (in minutes) to reach a detection threshold. The relation between these two is indirect and complicated. However, the reviewer has a valid point in pointing to a discussion about the overlap and the comparison of sensitivities between the two methods. Our additional analyses have been helpful in further characterization of RT-LAMP and we thank the reviewer in pointing us in that direction. These data are now included in the revised manuscript. Specifically, we have added:

1. Supplementary Figures 13– Showing the overlap analysis for mFISH and RT-LAMP experiments.

2. Supplementary note 4 and a section titled "mRNA FISH and microchip amplification overlap analysis" in the methods – These discuss the details about the overlap analysis between RT-LAMP and FISH, and the formulations used.

3. In the revised manuscript Figure 6b, we converted interpreted intensities in the RT-LAMP from [minutes] to [36 - minutes] (where 36 was the total RT-LAMP reaction time). This transformation allowed a more accurate presentation of the concordance between mFISH and RT-LAMP. This figure has been shown below in this document as **Response Figure 1**. Also, to allow comparison, the overlap analysis for 2 serial mRNA FISH sections has been shown as **Response Figure 2** in this document.

Please consider 2 other comments when comparing FISH with RT-LAMP in a quantitative analysis.

A. The grid placement and averaging done to visualize the FISH data as a pixelated graph artificially increases the FISH sensitivity in terms of number of positive pixels. This is likely due to splitting of a FISH signal into more than 1 pixel (max 4) depending on the grid line placement.

B. The positive pixel ratio (Number of positive pixels in RT-LAMP / Number of positive pixels in FISH) which is indicative of relative sensitivity of the 2 techniques was 0.92. The overlap bar graph demonstrates comparable sensitivities of the 2 techniques.

<u>Reviewer's Comment:</u> As said in the rebuttal letter, mRNA FISH is more comparable to RT-LAMP than IHC would be. However, it would be nonetheless important to correlate TOP2A gene expression to IHC used in clinic.

<u>Our Response:</u> We have performed the comparison of TOP2A mRNA from our technique with IHC on adjacent sections. The results for these are presented in **Supplementary Fig. 14** (also shown as **Response Figure 3** in this document). As can be seen from the data, the pattern of expression was consistent between RT-LAMP and IHC. We have also added them in this response document below also (3rd Figure). These experiments provided additional validation of our technique and we thank the reviewer for steering us in that direction.

<u>Reviewer's Comment:</u> For this new experiment, it is not clear from the result section or the M&M section which of the list of probes in Sup Note 3 were used.

<u>Our Response:</u> We have used all the probes listed in **Supplementary Note 3** for the detection of TOP2A mRNA using the mRNA-FISH (mFISH) technique. The protocol for mFISH were standard from commercial Stellaris RNA FISH and are based on the previously published protocol. (https://www.biosearchtech.com/support/resources/stellaris-protocols) In short, multiple labeled oligonucleotide probes are hybridized to the same mRNA and the cumulative signal from all the probes is detected as a bright spot.

<u>Reviewer's Comment:</u> In Fig. 2 and Sup. Fig. 2 the reviewer does not see the changes made in the Threshold time axes.

<u>Our Response:</u> The reviewer is correct that there was an error in the axis labels of standard curve of **Fig. 2b** which has now been corrected in the paper. The updated figure has been added to the main manuscript (also shown in this response document as the last figure). In **Supplementary Fig. 2a**, the x-axis represents the threshold times. In **Supplementary Fig. 2b**, we changed the x-axis label from "Concentration factor of purified RNA from Tissue" to "Total RNA from purified RNA from Tissue (1x= 2450 ng)" as the reviewer had previously suggested. The new figures include R^2 values for all curves.

<u>Reviewer's Comment:</u> The new experiment shown in Fig2b is quite different from the previous one in the original version of the manuscript. This really questions the reproducibility of this experiment.

<u>Our Response:</u> We would like to point out that **Fig. 2b** is the off-chip controls (RT-PCR) and do not affect the on-chip experiments or their interpretation in any way. We repeated the experiments with lower primer concentrations and obtained better results which showed reduced amplification from the blanks. The new experiment is very similar to the previous experiment with very similar threshold times and amplification profiles. Both the previous and the new **Fig. 2b** are added below in this document

(**Response Figure 4 below for reviewer's consideration**) for comparison. The only differences as can be seen from the figures were the truncated curves at cycle number 40. Moreover, there was non-specific amplification from the no-template control in the previous **Fig. 2b** which was resolved in the new experiment presented in the paper. To achieve this, titration of primer concentration was performed and finally a reduced forward and reverse primer concentration of 100nM (compared to 200nM previously) per reaction gave the best results. This primer titration was recommended by the manufacturer of RT-PCR kit (RNA ultrasensitive Thermofisher) to reduce non-specific amplification as a part of reaction optimization. The methods section have been updated with the new primer concentrations.

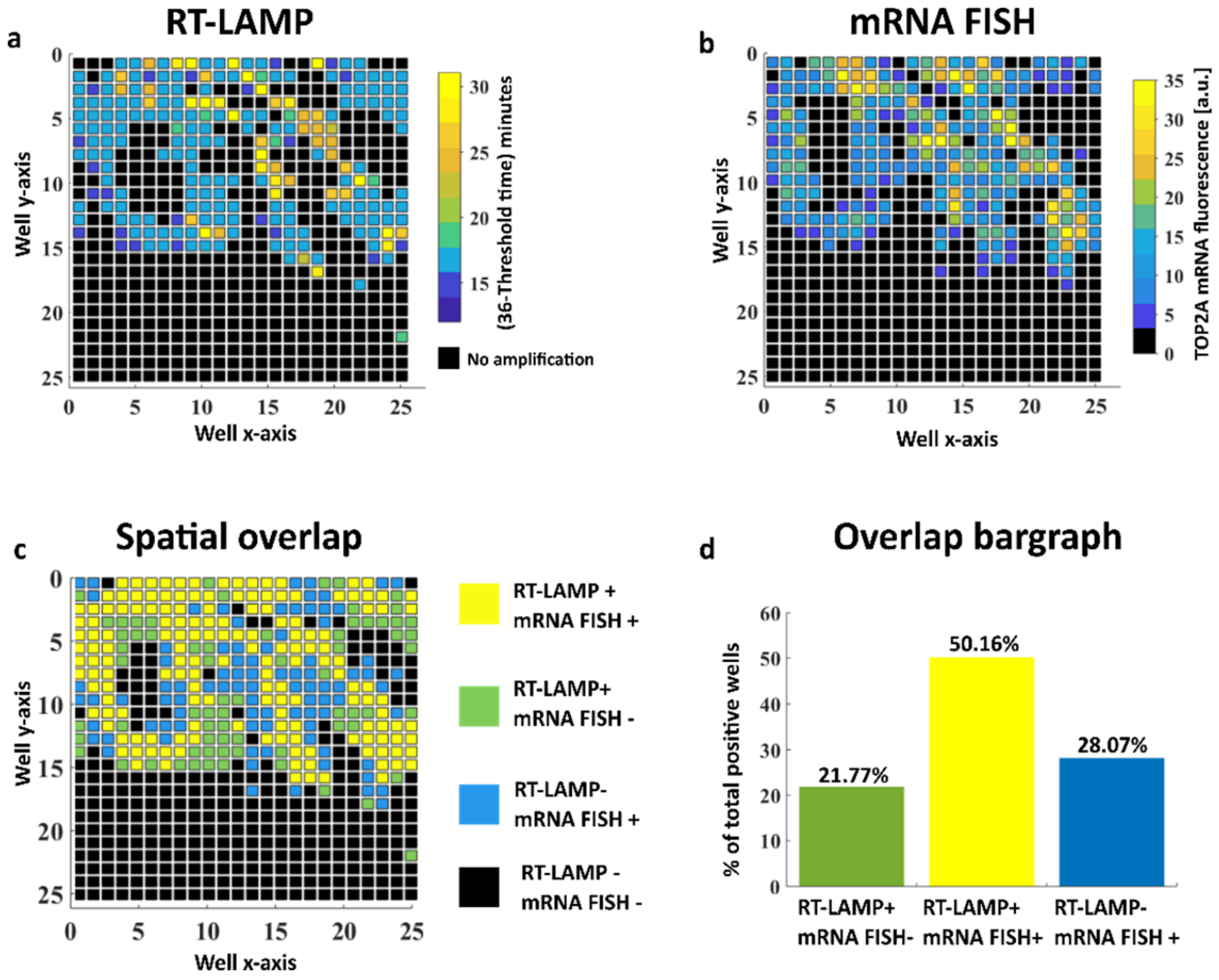
a
RT-LAMP
Well y-axis
Well x-axis
(36-Threshold time) minutes
No amplification
b
mRNA FISH
TOP2A mRNA fluorescence [a.u.]
c
Spatial overlap
RT-LAMP + mRNA FISH +
RT-LAMP+ mRNA FISH -
RT-LAMP- mRNA FISH +
RT-LAMP - mRNA FISH -
d
Overlap bargraph
% of total positive wells
21.77%
50.16%
28.07%
RT-LAMP+ mRNA FISH-
RT-LAMP+ mRNA FISH+
RT-LAMP- mRNA FISH +

Response Figure 1 | RT-LAMP - mRNA FISH overlap analysis. a, spatial threshold map using RT-LAMP. **b,** pixelated mRNA FISH intensity map **c,** Spatial binary overlap maps of the on-chip RT-LAMP with the mRNA FISH data. The binary overlap map denotes pixels with (i) both RT-LAMP and mRNA FISH signal (yellow), (ii) only RT-LAMP signal (green), (iii) only mRNA FISH signal (light blue), and (iv) neither RT-LAMP nor mRNA FISH signal (black). **d,** bar graph showing the different percentages of positive pixels with (i) RT-LAMP signal only (green), (ii) both mRNA FISH and RT-LAMP signal, and (iii) only mRNA FISH signal (light blue). The graph shows that the two techniques have comparable sensitivities.

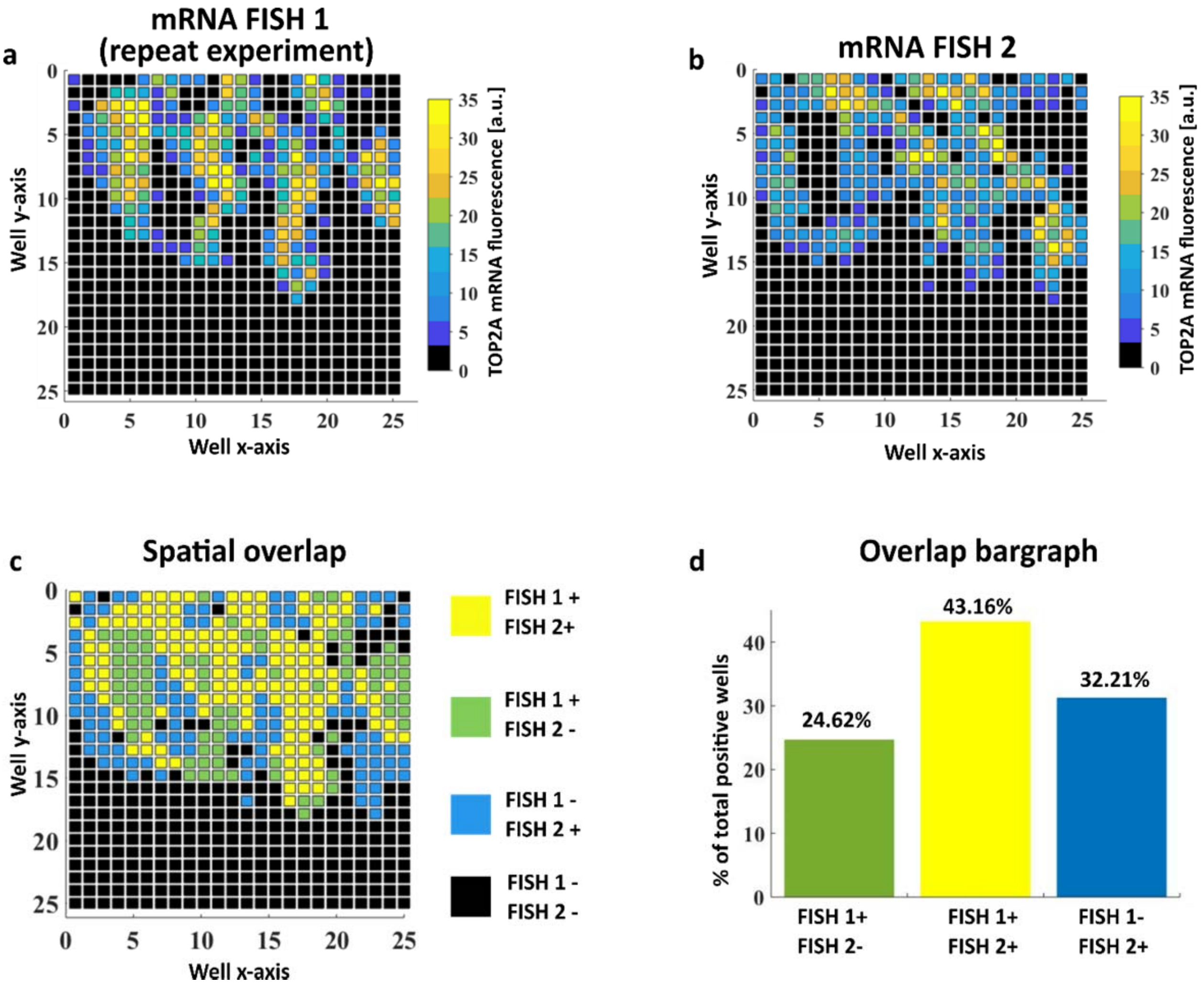

Response Figure 2 | mRNA FISH overlap analysis for adjacent sections from old Fig. 6 and Supplementary Fig. 12. a-b, mRNA FISH pixelated intensity map of experimental repeats 1 and 2. The mRNA FISH 1 (repeat FISH) image was resized to the same size as mRNA FISH 2 for overlap analysis. **c,** Spatial binary overlap maps of the two mRNA FISH data. The binary overlap map denotes pixels with (i) both section 1 and 2 mRNA FISH signal (yellow), (ii) only mRNA FISH section 1 signal (green), (iii) only mRNA FISH section 2 signal (light blue), and (iv) no mRNA FISH signal. (black). **d,** bar-graph showing the different percentages of positive pixels from (i) only mRNA FISH 1 (green), (ii) both mRNA FISH 1 and 2, and (iii) only mRNA FISH 2 (light blue). The positive pixel ratio (Total positive Fish 1 (repeat FISH)/Total positive Fish 2) between the 2 sections was 0.89, which was very similar to the 0.92 value obtained between RT-LAMP and FISH. Note that for our overlap analysis between RT-LAMP and mRNA FISH (Response Figure 1 above) we used the mRNA FISH section (mRNA FISH 2 in the figure above) with the higher number of positive pixels.

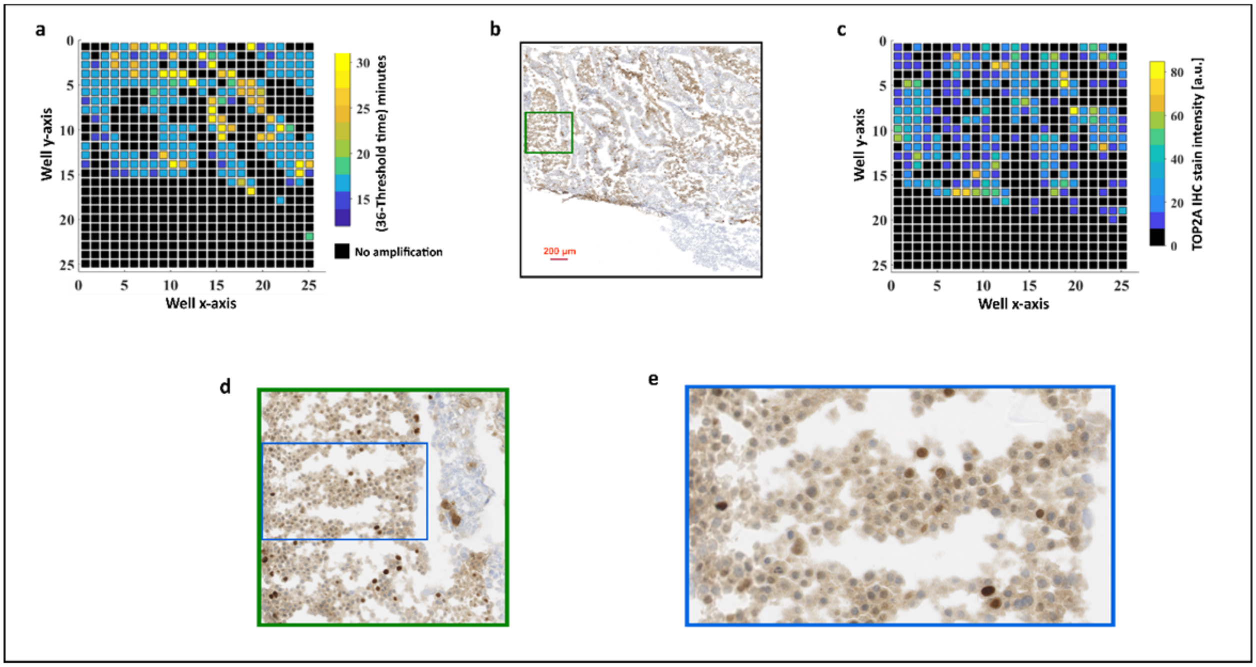

Response Figure 3 | Comparison of TOP2A mRNA expression through RT-LAMP with TOP2A IHC for adjacent sections. The RT-LAMP experiment is the same as shown in Figure 6 and IHC was performed on a serial section. **a,** Spatial threshold analysis from RT-LAMP. **b,** TOP2A IHC from adjacent sections in the region of interest. **c,** Pixelated TOP2A IHC expression obtained from “b”. The spatial signatures observed in the IHC images is consistent with those observed in RT-LAMP spatial threshold map. **d-e,** Zoomed in image for IHC showing staining.

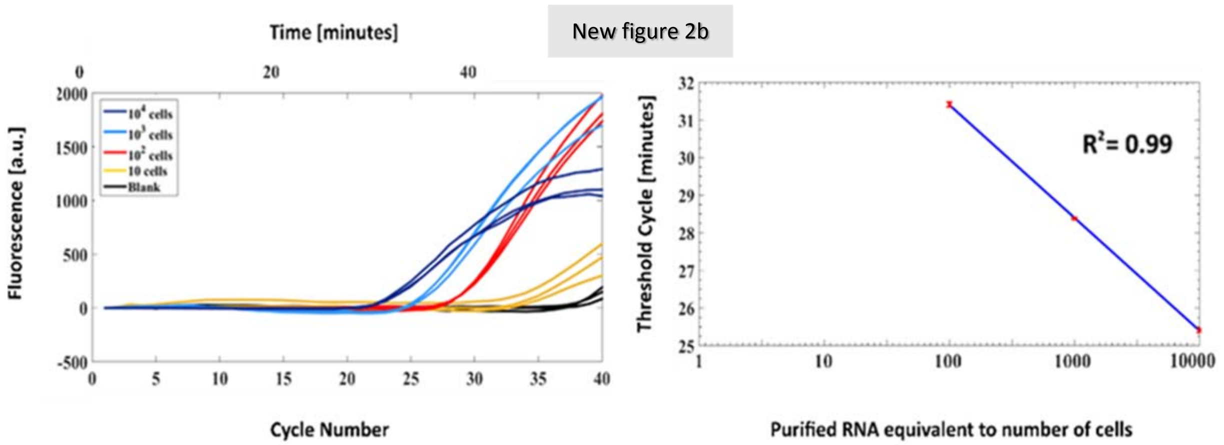

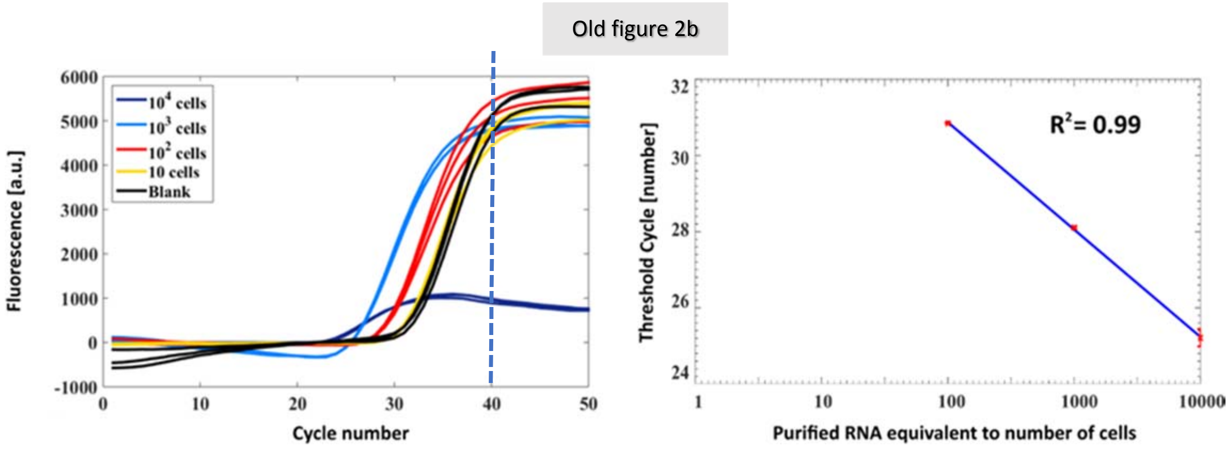

Response Figure 4 | Off-chip RT-PCR experiment comparison. The threshold times for both the new and old experiments were found to be very similar. Baseline fluorescence for the new experiment was corrected and the amplification curves were truncated at Cycle 40. No non-specific amplification from the negative control was observed in the new experiment.

REVIEWERS' COMMENTS:

Reviewer #2 (Remarks to the Author):

The second revision of the manuscript of Ganguli et al. now clarifies several points raised in the second review.

Concerning the correlation between RT-LAMP and FISH, the authors have now addressed this question adequately showing that the overlap between RT-LAMP positive wells and FISH positive wells is 50%. They also highlight similar sensitivity of the two techniques while lower duration of RT-LAMP over FISH.

Concerning the comparison between RT-LAMP and IHC the authors have now addressed this question convincingly using adjacent sections. A value for the overlap of positive wells as in Sup Fig.13d would bring a quantified answer. Figure legend of Sup Fig.14 does not include the description of d and e (unlike in response Figure 3). There are no scale bars in Fig.6d and e.

REVIEWERS' COMMENTS:

Reviewer #2 (Remarks to the Author):

1. The second revision of the manuscript of Ganguli et al. now clarifies several points raised in the second review. Concerning the correlation between RT-LAMP and FISH, the authors have now addressed this question adequately showing that the overlap between RT-LAMP positive wells and FISH positive wells is 50%. They also highlight similar sensitivity of the two techniques while lower duration of RT-LAMP over FISH. Concerning the comparison between RT-LAMP and IHC the authors have now addressed this question convincingly using adjacent sections.

A value for the overlap of positive wells as in Sup Fig.13d would bring a quantified answer.

Response- We thank the reviewer for the comment. The overlap analysis between RT-LAMP and IHC has now been added to the updated Supplementary Figure 14 (f-g). The overlap value obtained was 52% which is very similar to the value obtained for RT-LAMP and FISH overlap (50.16%).

Figure legend of Sup Fig.14 does not include the description of d and e (unlike in response Figure 3). There are no scale bars in Fig.6d and e.

Response- We thank the reviewer for the comment. The Figure caption for Sup. Fig. 14 has been updated to include the description of “d” and “e”, along with the additional overlap analysis figures “f” and “g”. The scale bars are present for Figure 6 a and c. Figures 6b, 6d are distribution heat maps calculated from the Figures 6a and c. There is no figure 6e.

The text “For figures b and d, each pixel is 100um*100um” is added in the caption of Figure 6.
